# Synthesis of palladium nanoparticles stabilized on Schiff base-modified ZnO particles as a nanoscale catalyst for the phosphine-free Heck coupling reaction and 4-nitrophenol reduction

**DOI:** 10.1038/s41598-023-38898-w

**Published:** 2023-07-25

**Authors:** Nuray Yılmaz Baran, Talat Baran, Mahmoud Nasrollahzadeh

**Affiliations:** 1 Department of Chemistry Technology, Technical Vocational School, Aksaray University, 68100 Aksaray, Turkey; 2 Department of Chemistry, Faculty of Science and Letters, Aksaray University, 68100 Aksaray, Turkey; 3 Department of Chemistry, Faculty of Science, University of Qom, Qom, PO Box 37185‑359, Iran

**Keywords:** Nanoscale devices, Nanoscale materials

## Abstract

Recently, the development of heterogeneous nanocatalytic systems using solid supports has been gaining importance due to some advantages such as easy handling, high thermal stability, high efficiency, reusability, and so on. Therefore, the design of catalyst supports for the preparation of stable heterogeneous catalytic systems is of great importance. In this work, Schiff base-modified ZnO particles have been developed (ZnO–Scb) as a novel support. A heterogeneous nanocatalyst system has then been prepared by immobilizing palladium nanoparticles (Pd NPs) on the ZnO-Scb surface as the support. The resulting palladium nanocatalyst (Pd–ZnO–Scb) structure has been characterized by different analytical techniques (FT-IR, XRD, TEM, FE-SEM, elemental mapping and EDS) and used to catalyze the Heck coupling reactions and 4-nitrophenol (4-NP) reduction. Test results revealed that Pd–ZnO–Scb could effectively couple various aryl halides with styrene in yields of up to 98% in short reaction times. Pd–ZnO–Scb was also efficiently used in the complete 4-NP reduction within 135 s at room temperature. Additionally, it was found that Pd–ZnO–Scb was more effective than other reported catalysts in the Heck coupling reaction. Moreover, the recycling tests indicated that Pd–ZnO–Scb could be easily isolated from the reaction medium and reused in seven consecutive catalytic runs while retaining its nanostructure.

## Introduction

Pd-catalyzed Heck coupling reaction is one of the significant synthetic methodologies used to form C–C bonds between olefins and aryl halides and has thus been widely used in the fabrication of various compounds such as natural products, pharmaceuticals, and bioactive materials^1–4^. Generally, homogeneous phosphine-based catalysts have played an important role in the Heck coupling reaction^3,5,6^. However, there are some problems such as the cost of most phosphine ligands, their toxicity, and the incapability of the catalyst to be readily recycled from the media, which limits their large-scale applications^7,8^. Therefore, efforts to substitute phosphine-based catalysts with heterogeneous solid-supported Pd catalysts have been gaining much interest recently. Different organic/inorganic compounds such as zeolites, carbon materials, metal oxides, MOFs, silica, and polymers have been utilized as carriers to prepare Pd catalysts with high activity and recyclability^9–14^. However, many developed heterogeneous catalysts have poor recyclability and chemical stability, which restrict their application in industrial or academic fields^15^. The selection or design of suitable supports can help overcome these problems. Moreover, the development of environmentally friendly, economic, highly stable, and recyclable catalytic systems is needed for the Heck coupling reaction from the green chemistry aspect.

Removal of toxic inorganic/organic contaminants from wastewater and soil is a serious and imperative challenge for human life^16–19^. Nitrophenols with high risks to the humans and environment are one of the most significant major organic pollutants in agricultural and industrial wastewaters due to their highly toxic nature, stability, and solubility in water^20^. In particular, 4-NP is an infamous organic pollutant commonly found in industrial wastewaters^21^. When it is discharged into the environment, it remains for a long time due to its high stability, thus negatively affecting human health, the environment, and aquatic life^22^. Therefore, 4-NP removal from water/wastewater is of great importance. Various conventional methods such as adsorption, ion exchange, oxidation, reverse osmosis, and membrane separation have previously been applied for the removal of this contaminant^23–25^. However, the treatment or purification of water/wastewater by these methods is not generally sufficient due to some weaknesses such as low adaptability, extraordinary cost, low efficiency, and generation of contamination^26,27^. Among these processes, catalytic reduction is highly effective due to its simplicity, inexpensiveness, formation of non-toxic compounds, speed, and simplicity of operation^28,29^. Additionally, reduction products, which are obtained as a result of the catalytic reduction, are valuable compounds for industrial applications. For example, 4-NP reduction product, 4-aminophenol (4-AP), has a significant role in the fabrication of analgesic and antipyretic drugs^30–32^. Therefore, catalytic reduction is a good strategy and the development of highly active catalysts is important.

Recently, the preparation of nanosized metal oxides has gained growing interest from researchers due to their optical, electrical, biological, and catalytic prowess^33,34^. Therefore, different metal oxides have been designed and used in various fields including water treatment, solar cells, biomedicine, and catalysis^35,36^. Among metal oxide NPs, ZnO is one of the most effective compounds widely used in various applications due to its reasonable cost, simple fabrication, biocompatibility, high availability, and non-toxicity^37,38^. Additionally, ZnO particle surfaces can be easily modified with different functionalities. For example, (3-aminopropyl)triethoxysilane (APTES), which is a member of the organosilanes, is a good agent to provide free –NH_2_ groups on the surface of ZnO^39,40^. According to the literature, Schiff base ligands are able to coordinate many different metals and metal oxides and can act as catalysts for various organic transformations and different applications^41–43^. In this regard, reactive NH_2_ groups on the ZnO surface can be further modified by reacting with aldehydes or ketones to form Schiff bases, which strongly interact with metal ions. Schiff base-modified ZnO particles can act as good catalyst supports for the synthesis of different catalysts and the resulting catalysts can be used in different industrial or academic applications.

**Scheme 1:**
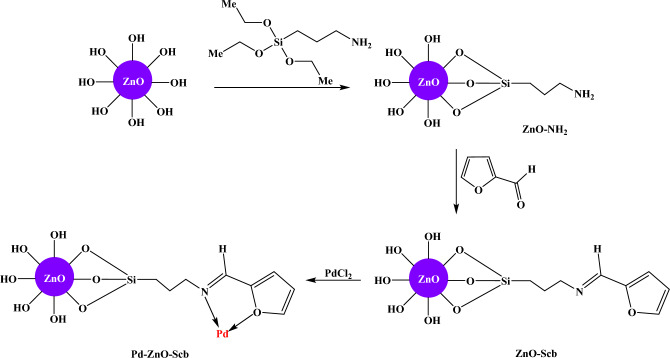
Schematic representation of the synthesis of Pd–ZnO–Scb nanocatalyst.

In this work, Schiff base-modified ZnO particles have been designed as novel catalyst supports for the first time. Pd nanocatalysts have then been synthesized via the stabilization of Pd on the developed support (Scheme 1). The structure of Pd–ZnO–Scb nanocatalyst has been characterized by different spectroscopic techniques such as FT-IR, FE-SEM, TEM, elemental mapping, EDS, and XRD. Characterization studies proved that Pd–ZnO–Scb was spherically shaped with an average particle size of 14 nm. The catalytic activity of Pd–ZnO–Scb was then evaluated in both the Heck coupling reaction and 4-NP reduction. Pd–ZnO–Scb catalyst successfully converted aryl halides to the corresponding Heck coupling products in yields of up to 98% in short reaction times. Additionally, Pd–ZnO–Scb catalyst displayed good performance by reduction of 4-NP within 135 s. Moreover, Pd–ZnO–Scb was easily isolated and reused for up to seven successive runs without changing its morphology and shape.

## Experimental

### Apparatuses and chemicals

All chemical compounds were purchased from Sigma-Aldrich Chemical Co. FT-IR spectra of ZnO, ZnO–NH_2_, and ZnO–Scb were recorded using a Perkin Elmer 100 FT-IR spectrophotometer. XRD spectra of ZnO and Pd–ZnO–Scb were recorded using a Rikagu smart lab system. FE-SEM/EDS images of ZnO, ZnO–NH_2_, ZnO–Scb, and Pd–ZnO–Scb were obtained using a QUANTA–FEG 250ESEM/EDAX–Metek instrument. TEM analysis of Pd–ZnO–Scb nanocatalyst was carried out using a Zeiss Sigma 300 instrument. NMR analyses were performed by a Bruker Avance III 400 MHz spectrometer. The 4-NP reduction was followed by UV–Vis spectroscopy (Genesys 10 S UV–Vis spectrophotometer).

### Synthesis of ZnO particles

Zn(NO_3_)_2_·6H_2_O (5.94 g) and (NH_4_)_2_CO_3_ (3.61 g) were separately dissolved in 20 mL of water to form solutions 1 and 2. Solutions 1 and 2 were next mixed and vigorously stirred at room temperature for 3 h to yield ZnO precursors. Finally, the resulting white precipitates were collected by filtration, rinsed with water, dried at 100 °C for 6 h and then calcinated at 550 °C for 4 h.

### Synthesis of APTES-functionalized ZnO (ZnO–NH_2_)

APTES-functionalized ZnO was prepared by heating ZnO (2 g) and APTES (8 mL) in 40 mL of anhydrous toluene for 48 h at 100 °C. Finally, ZnO–NH_2_ was filtered, washed with EtOH, and dried at 70 °C.

### Synthesis of Schiff base-modified ZnO (ZnO–Scb)

ZnO–NH_2_ (1.5 g) was dispersed in EtOH by sonication for 1 h, followed by the addition of 5 mL of furfural and refluxing the resulting mixture for 72 h. After the reaction was completed, the product denoted as ZnO–Scb, was collected by filtration, rinsed with hot EtOH, and dried at 60 °C.

### Decoration of Pd NPs on ZnO–Scb surface (Pd–ZnO–Scb)

1 g of ZnO–Scb and 0.2 g of PdCl_2_ were added to EtOH (30 mL) and the resulting mixture was heated at 70 °C. After 1 h, the reaction solution turned black, confirming the formation of Pd NPs on the support surface. After another 1 h, the dark-colored Pd–ZnO–Scb catalyst was isolated by filtration, washed with water and EtOH, and then dried at 60 °C.

### Pd–ZnO–Scb-catalyzed Heck coupling reaction

A mixture of aryl halide (1 mmol), styrene (1.5 mmol), Na_2_CO_3_ (2 mmol), and Pd–ZnO–Scb nanocatalyst (15 mg) in 4 mL of DMF was heated at 120 °C for the required time. At the end of the reaction (monitored by TLC), Pd–ZnO–Scb was recovered from the media and the mixture was extracted with CH_2_Cl_2_:H_2_O (1:1) three times. The organic phase bearing the Heck products was dried on MgSO_4_ and the products were obtained by evaporating the solvent. For further purification, column chromatography was carried out.

### Pd–ZnO–Scb-catalyzed 4-NP reduction

The mixture of an aqueous solution of 4-NP (2 mL, 1.5 × 10^–4^ M) and NaBH_4_ (0.5 mL, 0.05 M) was stirred for 2 min at room temperature. 10 mg of Pd–ZnO–Scb were then transferred into the media to start the catalytic reduction of 4-NP and the resulting mixture was stirred for the desired time. The progress of 4-NP reduction was followed by UV–Vis spectroscopy.

### NMR data of Heck coupling reaction products

#### (E)-1,2-Diphenylethene

^1^H NMR (400 MHz, CDCl_3_): δ_H_ = 7.51 (d, 4H), 7.34 (t, 4H), 7.27–7.24 (m, 2H), 7.11 (s, 2H); ^13^C NMR (100 MHz, CDCl_3_): δ_C_ = 137.40, 128.76, 128.70, 127.64, 126.54.

#### (E)-1-Methoxy-4-styrylbenzene

^1^H NMR (400 MHz, CDCl_3_): δ_H_ = 7.49–7.43 (m, 4H), 7.33 (t, 2H), 7.24–7.20 (m, 1H), 7.06 (d, 1H), 6.96 (d, 1H), 6.88 (d, 2H), 3.80 (s, 3H); ^13^C NMR (100 MHz, CDCl_3_): δ_C_ = 159.40, 137.74, 130.24, 128.68, 128.30, 127.77, 127.25, 126.70, 126.31, 114.21, 55.35.

#### (E)-1-Methyl-4-styrylbenzene

^1^H NMR (400 MHz, CDCl_3_): δ_H_ = 7.50 (d, 2H), 7.41 (d, 2H), 7.34 (t, 2H), 7.26–7.22 (m, 1H), 7.16 (d, 2H), 7.09 (d, 1H), 7.05 (d, 1H), 2.35 (s, 3H); ^13^C NMR (100 MHz, CDCl_3_): δ_C_ = 137.58, 134.62, 129.41, 128.66, 128.66, 127.77, 127.41, 126.45, 126.42, 21.25.

#### (E)-1-Nitro-4-styrylbenzene

^1^H NMR (400 MHz, CDCl_3_): δ_H_ = 8.21 (d, 2H), 7.62 (d, 2H), 7.55 (d, 2H), 7.42–7.31 (m, 3H), 7.26 (d, 1H), 7.13 (d, 1H); ^13^C NMR (100 MHz, CDCl_3_): δ_C_ = 146.85, 143.88, 136.24, 133.36, 128.92, 128.86, 127.05, 126.88, 126.33, 124.15.

#### (E)-4-Styrylbenzonitrile

^1^H NMR (400 MHz, CDCl_3_): δ_H_ = 7.64–7.57 (q, 4H), 7.53 (d, 2H), 7.39 (t, 2H), 7.33–7.30 (m, 1H), 7.21 (d, 1H), 7.08 (d, 1H); ^13^C NMR (100 MHz, CDCl_3_): δ_C_ = 141.89, 136.34, 132.50, 132.47, 128.87, 128.66, 126.94, 126.89, 126.77, 119.00, 110.66.

#### (E)-1-Nitro-3-styrylbenzene

^1^H NMR (400 MHz, CDCl_3_): δ_H_ = 8.36 (s, 1H), 8.09 (d, 1H), 7.79 (d, 1H), 7.55–7.49 (m, 3H), 7.39 (t, 2H), 7.32 (t, 1H), 7.23 (d, 1H), 7.12 (d, 1H); ^13^C NMR (100 MHz, CDCl_3_): δ_C_ = 148.81, 139.22, 136.32, 132.23, 131.83, 129.56, 128.87, 128.54, 126.86, 126.14, 122.03, 120.91.

### Supplementary data

Contains information about the NMR spectra of Heck coupling reaction products, ninhydrin color test of ZnO–NH_2_, FE-SEM images, elemental mapping and EDS spectra of samples, and also TEM image and XRD pattern of Pd–ZnO–Scb after seven cycles.

## Results and discussion

### Pd–ZnO–Scb characterization

**Figure 1:**
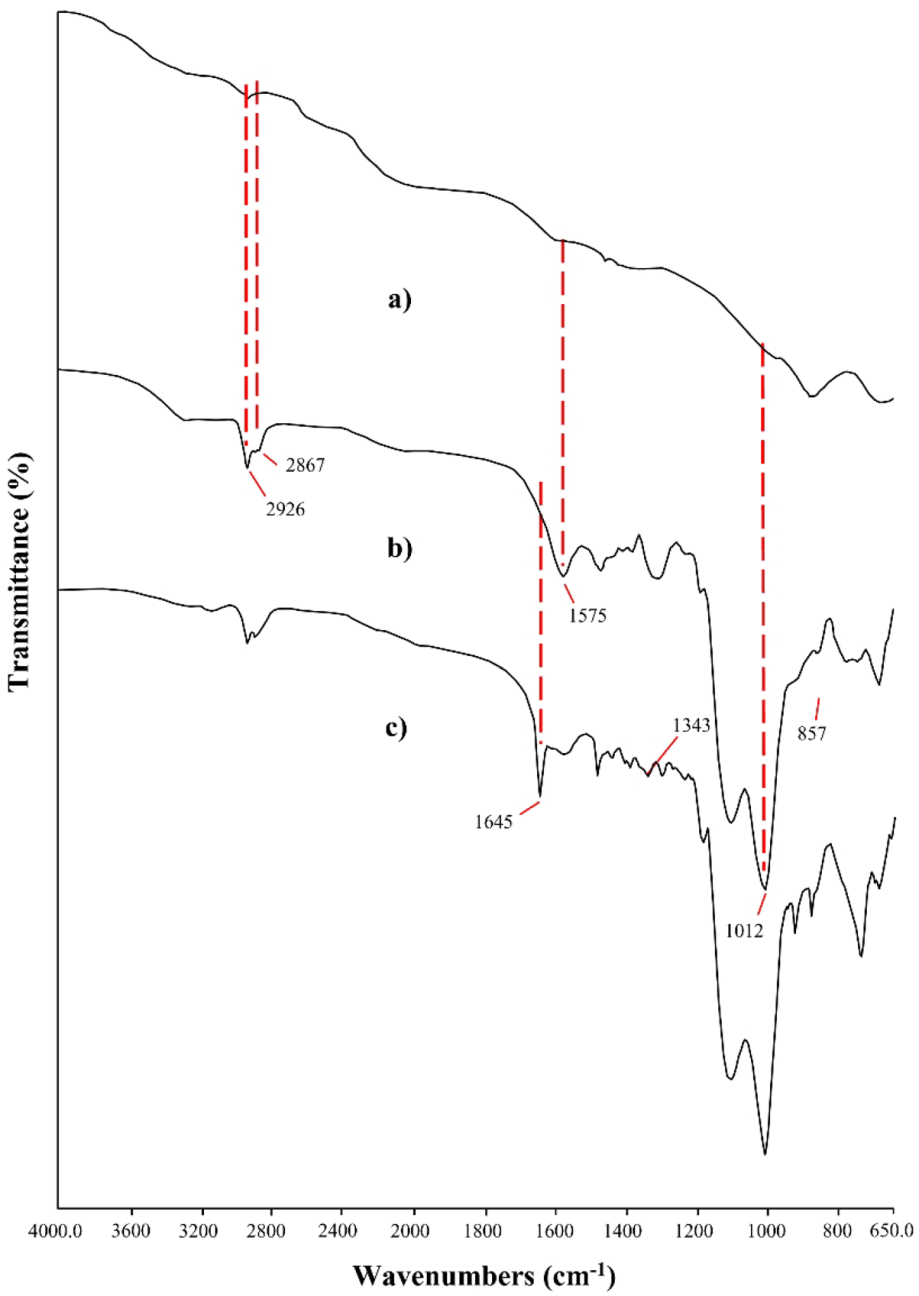
FT-IR spectra of ZnO (**a**), ZnO–NH_2_ (**b**), and ZnO–Scb (**c**).

The formation of ZnO, ZnO–NH_2_, and ZnO–Scb were confirmed by FT–IR analysis and the corresponding spectra are given in Fig. 1. Generally, ZnO displays an absorption band below 600 cm^−1^ related to the Zn–O stretching vibration^45^. However, this stretching band could not be detected due to the range of ATR/FT-IR. Therefore, XRD, EDS, and FE-SEM analyses were performed to study the fabrication of ZnO. Figure 1b shows the spectrum of ZnO–NH_2_. The strong peaks located at 2926 and 2867 cm^−1^ were assigned to the aliphatic C–H stretching vibrations of APTES molecules. Additionally, the peaks at 1575 and 1012 cm^−1^ were attributed to the NH_2_ deformation and Si–O stretching vibrations. These important and characteristic peaks confirmed the successful attachment of APTES to the surface of ZnO^46^. Additionally, a ninhydrin color test was performed to confirm the presence of APTES molecules on ZnO surface. For this purpose, 5 mg of ZnO–NH_2_ and 5 mg of ninhydrin were refluxed in 10 mL of ethanol^47^. After 3 min, the color of the suspension turned to purple, resulting in an absorbance peak of 580 nm in the UV–Vis spectrum (Figure S1). Based on the color test and UV–Vis analysis, APTES molecules are attached to the surface of ZnO. The peak at 1645 cm^−1^ in the FT-IR spectrum of ZnO–Scb indicated the presence of imine stretching vibration, confirming that the condensation reaction of ZnO–NH_2_ with furfural had been successfully performed.

**Figure 2:**
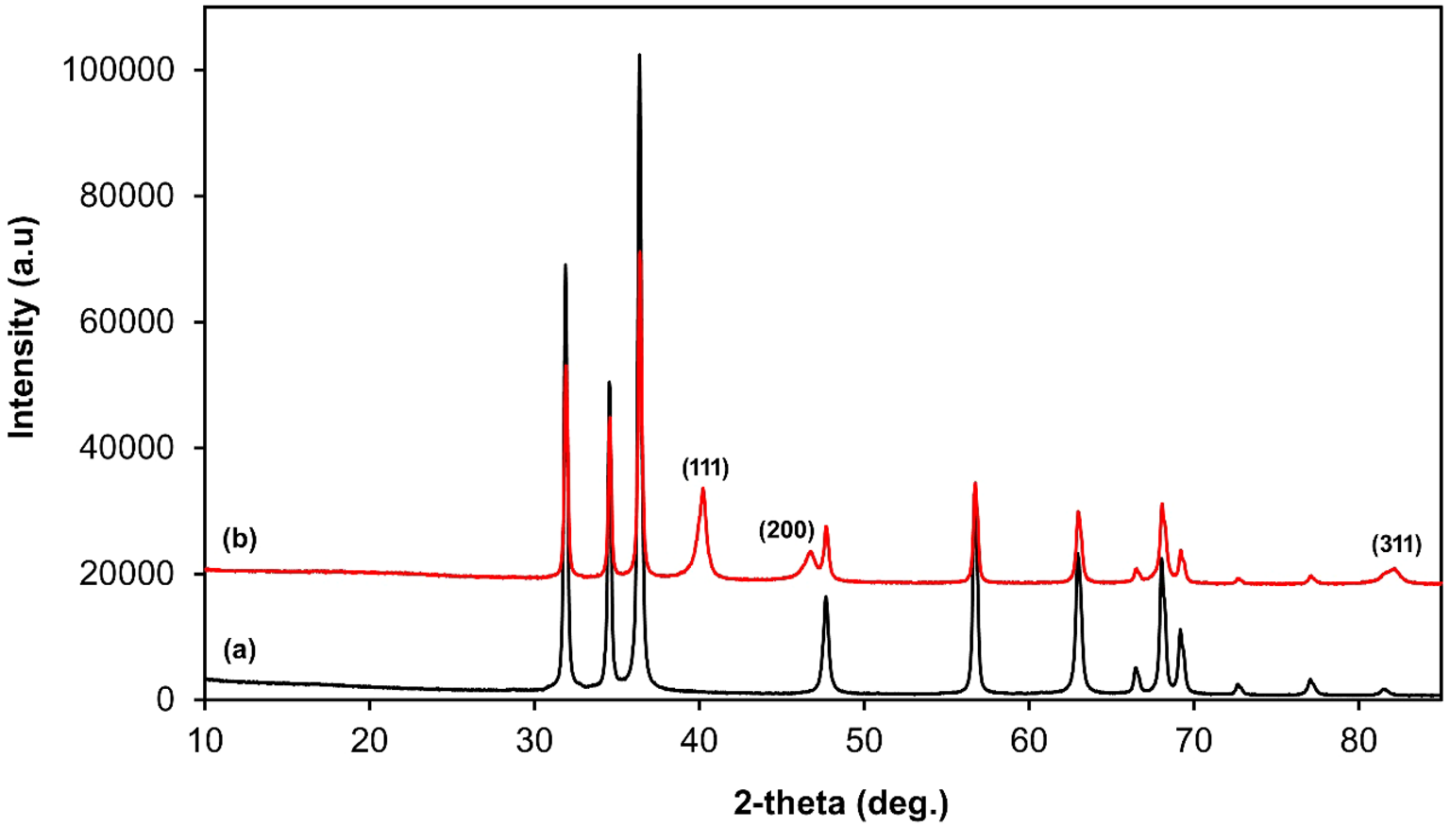
XRD patterns of ZnO (**a**) and Pd–ZnO–Scb (**b**).

The XRD patterns of ZnO and Pd–ZnO–Scb are shown in Fig. 2. In the XRD spectrum of ZnO, the peaks at 2θ values of 31.89°, 34.54°, 36.38°, 47.62°, 56.68°, 62.91°, 66.40°, 68.05°, and 69.18° were attributed to the (1 0 0), (0 0 2), (1 0 1), (1 0 2), (1 1 0), (1 0 3), (2 0 0), (1 1 2), and (2 0 1) planes of ZnO hexagonal zincite phase, respectively^48^. These results indicated the high purity of the prepared ZnO. Following the preparation of Pd NPs, ZnO peaks did not change while new peaks were observed at 40.16°, 46.60°, and 82.23°, which were associated with the (111), (200), and (220) planes of Pd, respectively, confirming the stabilization of Pd NPs on the support^49^.

To examine the morphological properties of synthesized ZnO, ZnO–NH_2_, ZnO–Scb, and Pd–ZnO–Scb, FE-SEM analysis was performed and the images are illustrated in Figure S2. As it can be observed in Figures S2a-c, ZnO NPs were aggregated and spherical. After the fabrication of ZnO–NH_2_ and ZnO–Scb, it was observed that ZnO surface was covered with particles, confirming the successful chemical modification of ZnO. The FE-SEM images of Pd–ZnO–Scb indicated the deposition of Pd NPs on ZnO–Scb surface with a nearly spherical shape. EDS analyses were performed to determine the presence of elements in ZnO, ZnO-NH_2_, and Pd-ZnO-Scb. Additionally, elemental mapping analysis was conducted to investigate the distribution of elements on the surface of the Pd-ZnO-Scb complex. The corresponding spectra are displayed in Figures S3 and S4, respectively. As observed in Figure S3, the EDS spectrum of ZnO showed peaks corresponding to Zn and O elements. The EDS spectrum of ZnO–NH_2_ displayed the presence of C, N, and Si peaks, related to APTES molecules, which confirmed that APTES molecules were attached to the surface of ZnO. As for Pd–ZnO–Scb spectrum, Pd peaks were observed in addition to the expected elements such as C, N, O, Si, and Zn. Additionally, the presence of Pd was determined using elemental mapping (Figure S4); which indicated the uniform dispersion of Pd on the ZnO–Scb surface.

**Figure 3:**
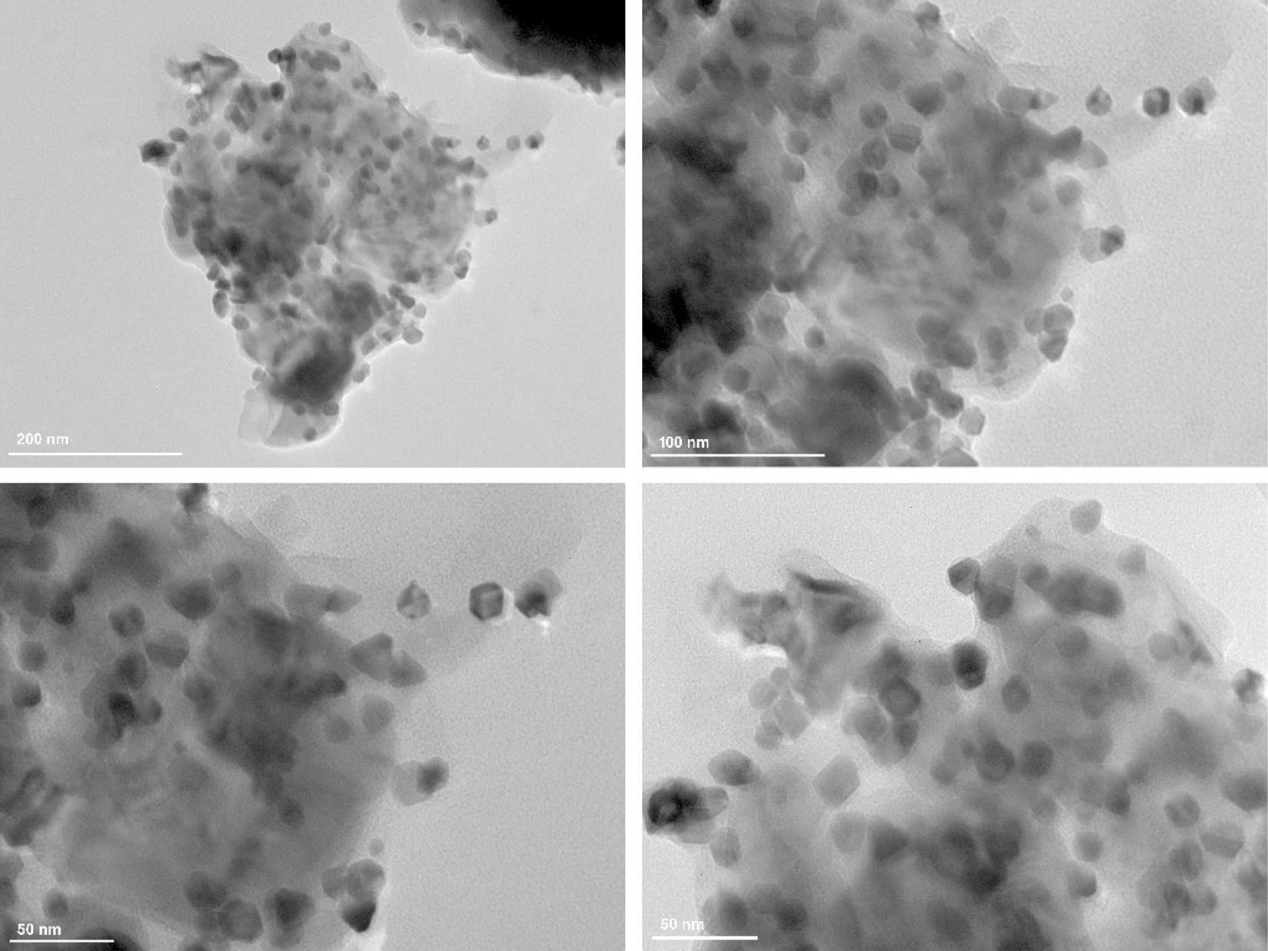
TEM images of Pd–ZnO–Scb.

**Figure 4:**
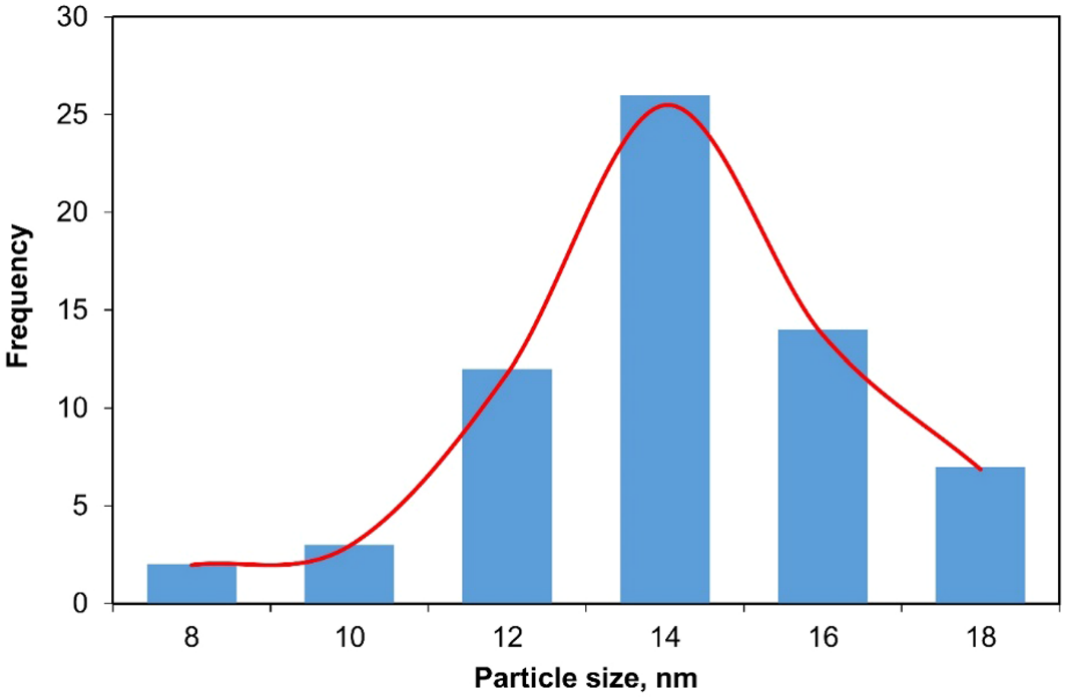
Particle size distribution of Pd–ZnO–Scb.

To further examine the shape and size of Pd–ZnO–Scb, TEM analysis was carried out (Fig. 3). The TEM images of Pd–ZnO–Scb clearly indicated the homogeneous dispersion of the Pd NPs, observed as spherical black spots, on its support. The average diameter of Pd NPs was about 14 nm (Fig. 4).

### Investigation of catalytic activity of Pd–ZnO–Scb

**Figure 5:**
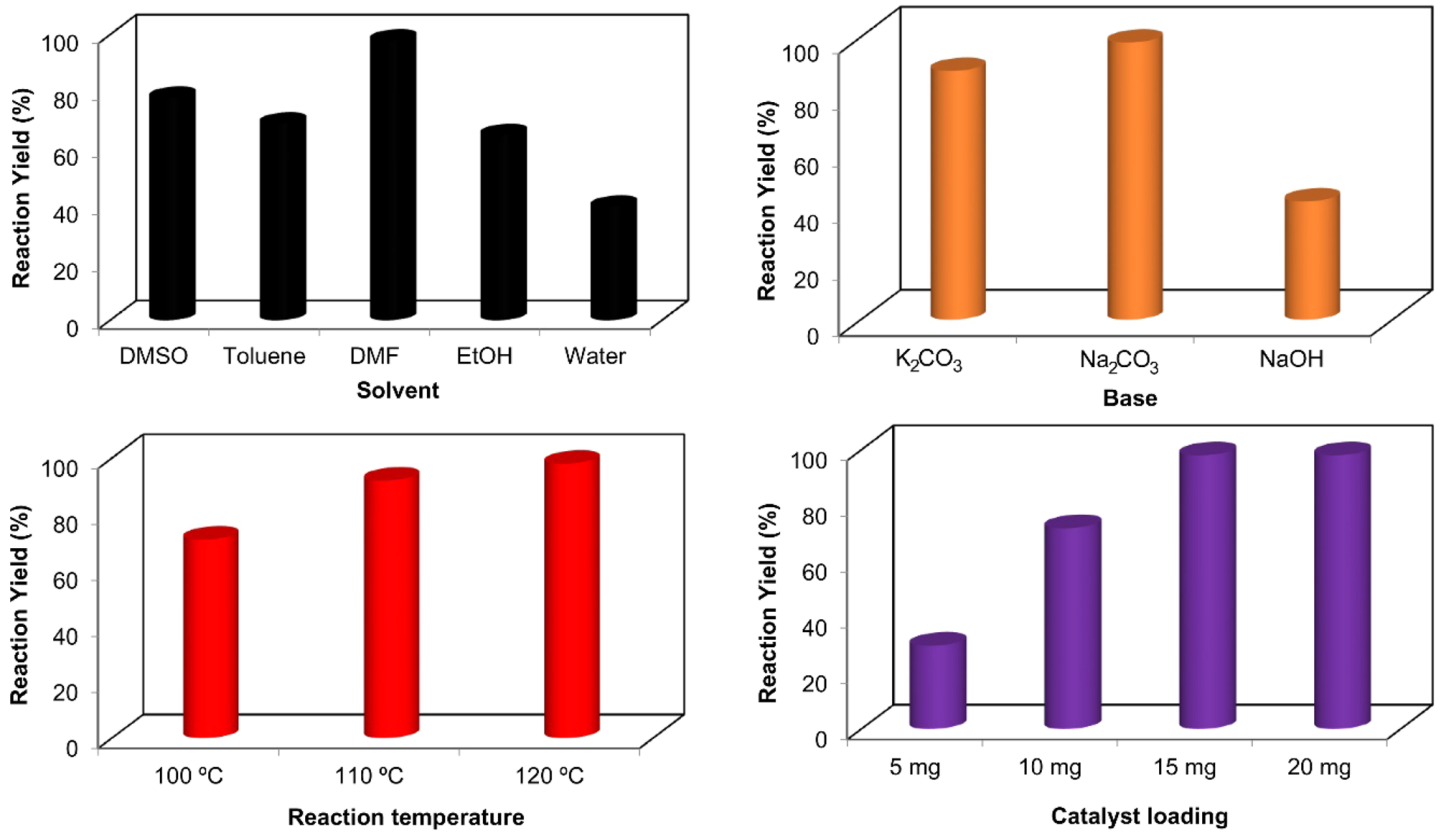
Determination of optimal reaction conditions for the reaction of 1-bromo-4-nitrobenzene and styrene in the presence of Pd–ZnO–Scb.

After complete characterization of Pd–ZnO–Scb, its catalytic prowess was evaluated in the Heck coupling reaction. In the early step, the reaction between styrene and 1-bromo-4-nitrobenzene was selected as a model reaction and the effects of time, solvent, base, and catalyst loading were then studied to determine the optimal conditions. As observed in Fig. 5, the optimal reaction conditions were 15 mg of Pd–ZnO–Scb, temperature of 120 °C, Na_2_CO_3_ as the base, and DMF as the solvent. Afterward, the generality and substrate tolerance of Pd–ZnO–Scb catalytic system was tested in the Heck coupling reaction of different substituted aryl halides under optimal reaction conditions and the results are shown in Table 1. The reaction of styrene and aryl iodides bearing different groups such as –OMe, –Me, and –NO_2_ was successfully performed with good reaction yields. For example, 4-iodoanisole was converted to the target product with 95% yield within 1.5 h. 1-Iodo-3-nitrobenzene was coupled with styrene with 92% yield. The catalytic potential of Pd–ZnO–Scb was also tested using different substituted aryl bromides and it was found that Pd–ZnO–Scb successfully catalyzed these reactions by providing good isolated yields. For example, 4-bromotoluene was reacted with styrene and the corresponding Heck product reached 91% yield within 3 h. Bromobenzene substrate formed the corresponding Heck product with 94% yield. The reaction of 4-bromobenzonitrile gave the desired product in 96% isolated yield in 1.5 h. The results obtained clearly showed that Pd–ZnO–Scb played a crucial role in the Heck coupling reactions. On the other hand, the catalytic performance of some previously reported catalysts in the Heck coupling reaction between 4-iodoanisole and styrene have been summarized to compare with that of our catalyst (Table 2). It is evident that Pd–ZnO–Scb performed better than the other catalysts in terms of reaction yield and time.

**Table 1:** Substrate scope for Pd–ZnO–Scb-catalyzed Heck coupling reaction.^a^


Entry	X	R	Time (h)	Yield (%)^b^
1	I	4-OMe	1.5	95
2	I	4-Me	2	93
3	I	3-NO_2_	2	92
4	Br	4-Me	3	91
5	Br	4-OMe	2	94
6	Br	4-CN	1.5	96
7	Br	4-NO_2_	1.5	98
8	Br	3-NO_2_	2.5	93
9	Br	H	2	94
10	Cl	4-OMe	5	75
11	Cl	4-Me	6	63

^a^Reaction conditions: Aryl halides (1 mmol), Na_2_CO_3_ (2 mmol), styrene (1.5 mmol), 15 mg of Pd–ZnO–Scb, and DMF (4 mL) at 120 °C.

^b^Isolated yield.

**Table 2:** Comparison of the catalytic activity of Pd-ZnO-Scb with reported catalysts in Heck coupling reaction between 4-iodoanisole and styrene.

Entry	Catalyst	Time (h)	Yield (%)	Ref
1	Pd NPs/nitrogen-doped mesoporous carbon	4	93	^50^
2	MCM-41-SH-Pd(0)	6	95	^51^
3	PVC-AE-Pd0	5	90	^52^
4	‘Si’-2As-Pd(0)	6	92	^53^
5	Polyphenol microsphere-supported Pd catalyst	7	90	^54^
6	ERGO–Pd	2	94	^55^
7	PIC–PdNPs	20	88	^56^
8	Pd(0)-Arg-boehmite	18	73	^57^
9	Polystyrene supported thiopseudourea Pd(II) complex	10	88	^58^
10	Pd NPs supported on poly(*N*-vinylpyrrolidone)-grafted silica	2.5	95	^59^
11	Pd–ZnO–Scb	1.5	95	Present study

**Figure 6:**
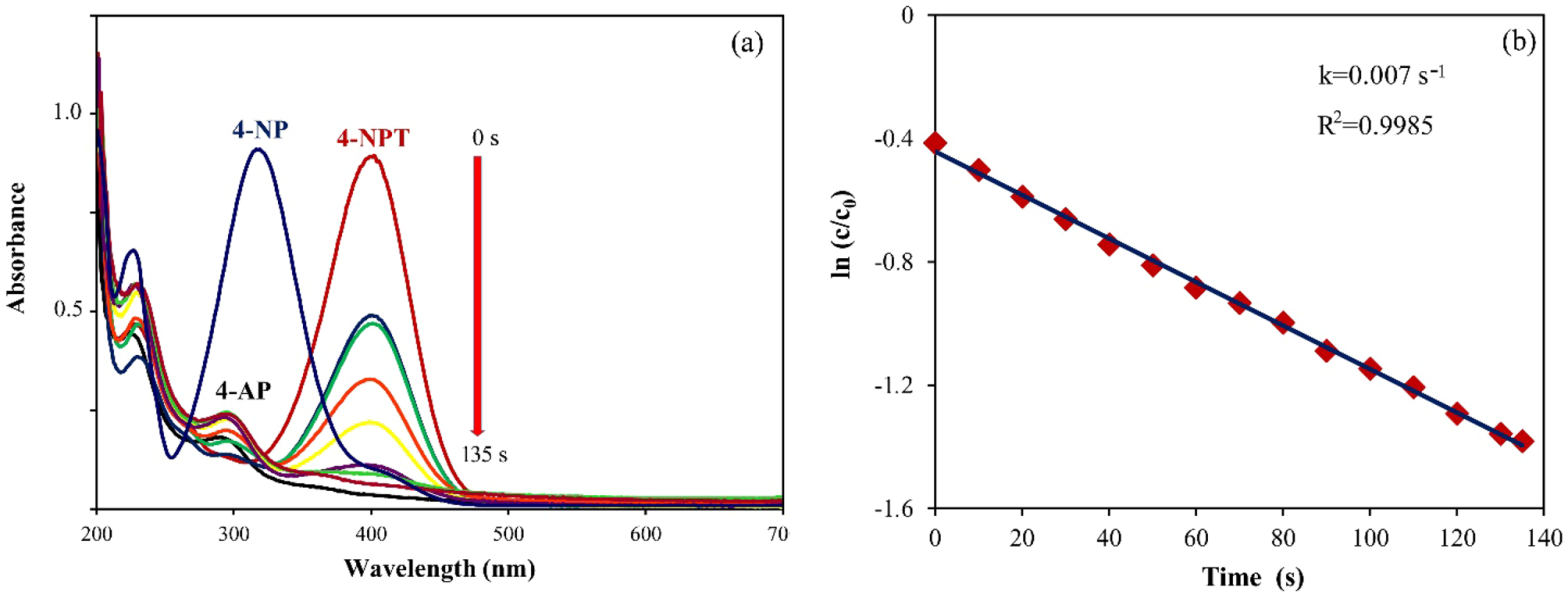
Reduction of 4-NP (**a**) and linear dependence graph between ln (c/c0) and time for 4-NP (**b**).

Inspired by the performance of our catalyst in the Heck coupling reaction, it was also used in the catalytic reduction of 4-NP to 4-AP by NaBH_4_. The reduction of 4-NP in the presence of a catalyst is simple compared to other methods due to the formation of merely one product (4-AP). In addition, the reaction progress can be easily followed by UV–Vis spectroscopy at 400 nm^60,61^. As Fig. 6a displays, 4-NP, which has a pale-yellow color, showed a maximum absorption at 317 nm in water solvent. Upon the addition of freshly prepared NaBH_4_ solution into 4-NP solution, the color of the solution changed to deep yellow and the absorption peak at 317 nm shifted to about 400 nm, indicating the formation of 4-nitrophenolate anion. Therefore, 4-NP reduction is carried out on 4-nitrophenolate anion (4-NPT). It was observed that this solution was very stable and the absorption peak at 400 nm did not change even after 5 h without Pd–ZnO–Scb. This indicated that Pd–ZnO–Scb was required for 4-NP reduction. Upon the addition of Pd–ZnO–Scb into 4-NP + NaBH_4_ mixture, the absorption at 400 nm gradually decreased and disappeared within 135 s. Additionally, a new peak appeared at about 300 nm simultaneously with the reduction reaction, confirming the formation of 4-AP. Moreover, the yellow color of the reaction solution turned colorless at the end of the catalytic reduction. All these findings confirmed the successful conversion of 4-NP to 4-AP by Pd–ZnO–Scb within 135 s without any side products, in concordance with previous studies. Figure 6b illustrates a linear correlation between ln (c/c_0_) and reaction time (t) for Pd–ZnO–Scb catalyzed 4-NP reduction. The rate constant was found as 0.007 s^−1^ for 4-NP reduction using the following equation. The catalytic system followed pseudo-first-order kinetics due to NaBH_4_ concentration being higher than 4-NP.

$${\text{ln }}\left( {{\text{c}}/{\text{c}}_{0} } \right) \, = \, - {\text{kt}}$$

where c_0_ and c are the initial and final concentrations of 4-NP at tested reaction time (t), respectively, and k (s^−1^) is the reaction rate.

Table 3 summarizes the comparison of the catalytic prowess of Pd–ZnO–Scb with those of various other catalysts in 4-NP reduction. The results showed that Pd–ZnO–Scb was the most active among the catalysts.

**Table 3:** Comparison of the catalytic activity of Pd-ZnO-Scb with those of other catalysts in 4-NP reduction.

Entry	Catalyst	Time	Ref
1	Au/PMMA	600 s	^62^
2	CuBDC•DMF	5 min	^63^
3	FPP/Au microspheres	13 min	^64^
4	AgNPs/ESM	900 s	^65^
5	AgNPs@MWCNTs-polymer composite	5 min	^66^
6	Ni-RGO	480 s	^67^
7	Pt/Fe_2_O_3_ MF	12 min	^68^
8	AgNPs@NC	8 min	^69^
9	Au@PZS@CNTs	16 min	^70^
10	RS–AgCl@Ag	4 min	^71^
11	Pd–ZnO–Scb	135 s	Present study

### Recyclability potential of Pd–ZnO–Scb

It is known that the recyclability of a catalyst is a crucial factor for both industrial and academic applications in terms of economy, cost-effectiveness, labor, and sustainability. Therefore, the recycling potential of Pd–ZnO–Scb nanocatalyst was investigated in the model Heck coupling reaction under optimized conditions. After each cycle, Pd–ZnO–Scb was isolated by filtration, rinsed with water and ethanol, and dried. The recovered Pd–ZnO–Scb was then directly used in the next reactions. The results revealed that Pd–ZnO–Scb could be reused and recycled up to 7 successive runs giving a product yield of 87%. To check the stability of Pd–ZnO–Scb, its surface was examined by TEM and XRD analyses following the seventh run and the corresponding images are given in Figures S5 and S6. It was found that the particle size, shape, and morphology of the recycled Pd–ZnO–Scb were almost identical to that of the fresh catalyst, indicating the structural stability of Pd–ZnO–Scb.

A hot filtration test was performed on 1-bromo-4-nitrobenzene under the optimal reaction conditions determined in the Heck coupling reaction. The Heck coupling reaction was carried out for 45 min in the presence of Pd–ZnO–Scb nanocatalyst. The catalyst was then recovered from the reaction medium and allowed to react for an additional 45 min under the same conditions to complete the reaction time with the filtrate. It was observed that the Heck coupling reaction did not progress further, indicating that Pd–ZnO–Scb did not leach. Inductively Coupled Plasma (ICP) analysis revealed that the content of Pd NPs loaded on the ZnO–Scb surface was about 9.6%. To check the heterogeneity of catalyst, which is an important factor, the phenomenon of leaching was studied by ICP analysis of the resulting reaction mixture. According to the ICP analysis, the Pd content of the used catalyst was determined as 9.2%.

## Conclusion

In summary, Pd–ZnO–Scb has been designed as a retrievable/recyclable heterogeneous nanocatalyst by stabilizing Pd NPs on the prepared Schiff base functionalized ZnO support. The nano-structured Pd–ZnO–Scb was successfully characterized by FT-IR, FE-SEM, TEM, XRD, elemental mapping, and EDS analyses. The catalytic prowess of Pd–ZnO–Scb was then evaluated in the Heck coupling reaction and 4-NP reduction. The results indicated that Pd–ZnO–Scb successfully coupled aryl chlorides, bromides and iodides with styrene, giving 63–98% isolated yields. 4-NP reduction was also efficiently catalyzed by Pd–ZnO–Scb in a short reaction time (135 s). Furthermore, Pd–ZnO–Scb was utilized for seven successive runs with 87% reaction yield and the protection of the nanostructure following the recycling tests was confirmed by TEM analysis. Due to its low cost, simplicity of separation, high yield, stability, and high recoverability/reusability, Pd–ZnO–Scb has a high potential for catalytic transformations and therefore, its catalytic prowess can be evaluated in other applications in the future.

## Supplementary Information

Supplementary Figures.

## Data Availability

All data generated or analyzed during this study are included in this published article and its supplementary information files.

## References

[1] Lee GS, Kim D, Hong SH (2021). Pd-catalyzed formal Mizoroki-Heck coupling of unactivated alkyl chlorides. Nat. Commun..

[2] Kantam ML, Annapurna M, Likhar PR, Srinivas P, Mirzadeh N, Bhargava SK (2013). Palladium complexes containing multidentate phenoxy-pyridyl-amidate ligands: Highly efficient catalyst for Heck coupling of deactivated aryl halides. J. Organomet. Chem..

[3] Wen X, Dai T, Zhao Z, Luo Z, Chen C, Sun W, Ran M (2020). In situ preparation of magnetic nickel-containing functionalized carbon nanotubes to support palladium as a catalyst for the Heck reaction. Appl. Catal. A Gen..

[4] Meng G, Szostak M (2015). General olefin synthesis by the palladium-catalyzed Heck reaction of amides: Sterically controlled chemoselective N-C activation. Angew. Chem..

[5] Mino T, Shirae Y, Sasai Y, Sakamoto M, Fujita T (2006). Phosphine-free palladium catalyzed Mizoroki-Heck reaction using hydrazone as a ligand. J. Org. Chem..

[6] Mansour W, Suleiman R, Iali W, Fettouhi M, El Ali B (2021). Synthesis, crystal structure, and catalytic activity of bridged-bis(N-heterocyclic carbene) palladium(II) complexes in selective Mizoroki-Heck cross-coupling reactions. Polyhedron.

[7] Cao N, Ran MF, Feng Y, Chu W, Jiang C, Sun W, Chen C (2020). Density functional theory study of N-doping effect on the stability and activity of Pd/NCNT catalysts for Heck reaction. Appl. Surf. Sci..

[8] Esmaeilpour M, Sardarian A, Javidi J (2016). Dendrimer-encapsulated Pd(0) nanoparticles immobilized on nanosilica as a highly active and recyclable catalyst for the copper-and phosphine-free Sonogashira-Hagihara coupling reactions in water. Catal. Sci. Technol..

[9] Yang H, Han X, Ma Z, Wang R, Liu J, Ji X (2010). Palladium-guanidine complex immobilized on SBA-16: A highly active and recyclable catalyst for Suzuki coupling and alcohol oxidation. Green Chem..

[10] Kitamura Y, Sakurai A, Udzu T, Maegawa T, Monguchi Y, Sajiki H (2007). Heterogeneous Pd/C-catalyzed ligand-free Suzuki-Miyaura coupling reaction using aryl boronic esters. Tetrahedron.

[11] Zhou A, Guo R-M, Zhou J, Dou Y, Chen Y, Li J-R (2018). Pd@ZIF-67 derived recyclable Pd-based catalysts with hierarchical pores for high-performance Heck reaction. ACS Sustain. Chem. Eng..

[12] Yu R, Liu R, Deng J, Ran M, Wang N, Chu W, He Z, Du Z, Jiang C, Sun W (2018). Pd nanoparticles immobilized on carbon nanotubes with a polyaniline coaxial coating for the Heck reaction: Coating thickness as the key factor influencing the efficiency and stability of the catalyst. Catal. Sci. Technol..

[13] Biffis A, Centomo P, Del Zotto A, Zecca M (2018). Mosslemin, Pd metal catalysts for cross-couplings and related reactions in the 21st century: A critical eeview. Chem. Rev..

[14] Zuo L, Yu S, Zhang R, Li H, Wu Y, Abiev R, Sun Z, Sun Z (2023). Tunning Pd-Cu-based catalytic oxygen carrier for intensifying low-temperature methanol reforming. J. Clean. Prod..

[15] Zhou J, Sun H, Xu C, Wang Z, Zhang H, Guo D, Zhang J, Ji X, Liu L, Ma J, Tong Z (2022). Palladium nanoparticles supported on α-zirconium phosphate nanosheets as a highly efficient heterogeneous catalyst for the Heck reaction. J. Taiwan Inst. Chem. Eng..

[16] Yu H, Zhu J, Qiao R, Zhao N, Zhao M, Kong L (2022). Facile preparation and controllable absorption of a composite based on PMo_12_/Ag nanoparticles: Photodegradation activity and mechanism. Chem. Sel..

[17] Dong H, Zou Y, Zhang K, Sun Y, Hui B, Yang D, Cai L, Li J (2023). Biomimetic design of wood carbon-based heterogeneous catalysts for enhanced organic pollutants degradation. Chem. Eng. J..

[18] Xia G, Zheng Y, Sun Z, Xia S, Ni Z, Yao J (2022). Fabrication of ZnAl-LDH mixed metal-oxide composites for photocatalytic degradation of 4-chlorophenol. Environ. Sci. Pollut. Res..

[19] Anis SM, Hashemi SH, Nasri A, Sajjadi M, Eslamipanah M, Jaleh B (2022). Decorated ZrO_2_ by Au nanoparticles as a potential nanocatalyst for the reduction of organic dyes in water. Inorg. Chem. Commun..

[20] Chang Y-C, Chen D-H (2009). Catalytic reduction of 4-nitrophenol by magnetically recoverable Au nanocatalyst. J. Hazard. Mater..

[21] He Y, Wang M, Mao X, Zhang M, Feng X, Ji Z, Li Y, Xiong Z, Xing Z, Hu J, Wu G (2022). Selective recovery of gold from e-waste with 3D hierarchical porous amidoximated fabrics and its application in the reduction of 4-nitrophenol. Radiat. Phys. Chem..

[22] Abdelhamid HN (2021). High performance and ultrafast reduction of 4-nitrophenol using metal-organic frameworks. J. Environ. Chem. Eng..

[23] Das TK, Das NC (2022). Advances on catalytic reduction of 4-nitrophenol by nanostructured materials as benchmark reaction. Int. Nano Lett..

[24] Zhang C, Zhang Y, Zhang Y, Huang X, Li Y, Cao J, Zhou C (2022). One-pot synthesis of ultrathin 1T-MoS_2_ nanosheets as efficient catalyst for reduction of 4-nitrophenol. Mater. Lett..

[25] Wu Y, Hao W, Li X, Qin L, Zhang T, Kang S-Z (2022). An efficient integrated catalyst for the reduction of 4-nitrophenol in a continuous flow system: Ag nanoparticles loaded graphene nanosheets immobilized on Ti meshes. Diam. Relat. Mater..

[26] Shamsollahi Z, Partovinia A (2019). Recent advances on pollutants removal by rice husk as a bio-based adsorbent: A critical review. J. Environ. Manag..

[27] Crini G (2006). Non-conventional low-cost adsorbents for dye removal: A review. Bioresour. Technol..

[28] Wang H, Ma Y, Shen Y, Zhang C, Yang J, Li P (2022). Designing of Co_3_O_4_/N-doped carbon and Co-Co_3_O_4_/N-doped carbon nanomaterials via sol-gel route with unexpected high catalytic performances toward hydrogenation reduction of 4-nitrophenol. J. Alloys Compd..

[29] Reddy Bogireddy NK, Mejia YR, Aminabhavi TM, Barba V, Becerra RH, Ariza Flores AD, Agarwal V (2022). The identification of byproducts from the catalytic reduction reaction of 4-nitrophenol to 4-aminophenol: A systematic spectroscopic study. J. Environ. Manag..

[30] Nemanashi M, Meijboom R (2013). Synthesis and characterization of Cu, Ag and Au dendrimer-encapsulated nanoparticles and their application in the reduction of 4-nitrophenol to 4-aminophenol. J. Colloid Interface Sci..

[31] Rode C, Vaidya M, Jaganathan R, Chaudhari R (2001). Hydrogenation of nitrobenzene to *p*-aminophenol in a four-phase reactor: Reaction kinetics and mass transfer effects. Chem. Eng. Sci..

[32] Guo M, He J, Li Y, Ma S, Sun X (2016). One-step synthesis of hollow porous gold nanoparticles with tunable particle size for the reduction of 4-nitrophenol. J. Hazard. Mater..

[33] Prochowicz D, Kornowicz A, Lewiński J (2017). Interactions of native cyclodextrins with metal ions and inorganic nanoparticles: fertile landscape for chemistry and materials science. Chem. Rev..

[34] Kamarajan G, Anburaj DB, Porkalai V, Muthuvel A, Nedunchezhian G (2022). Green synthesis of ZnO nanoparticles using *Acalypha indica* leaf extract and their photocatalyst degradation and antibacterial activity. J. Indian Chem. Soc..

[35] Muthuvel A, Said NM, Jothibas M, Gurushankar K, Mohana V (2021). Microwave-assisted green synthesis of nanoscaled titanium oxide: photocatalyst, antibacterial and antioxidant properties. J. Mater. Sci. Mater. Electron..

[36] Jayanthi PJ, Punithavathy IK, Jeyakumar SJ, Elavazhagan T, Muthuvel A, Jothibas M (2022). Influence of temperature on the structural, optical, morphological and antibacterial properties of biosynthesized silver nanoparticles. Nanotechnol. Environ. Eng..

[37] Raza A, Shoeb M, Mashkoor F, Rahaman S, Mobin M, Jeong C, Yusuf Ansari M, Ahmad A (2022). Phoenix dactylifera mediated green synthesis of Mn doped ZnO nanoparticles and its adsorption performance for methyl orange dye removal: A comparative study. Mater. Chem. Phys..

[38] Gea S, Situmorang SA, Pasaribu N, Piliang AFR, Attaurrazaq B, Sari RM, Pasaribu KM, Goutianos S (2022). Facile synthesis of ZnO-Ag nanocomposite supported by graphene oxide with stabilised band-gap and wider visible-light region for photocatalyst application. J. Mater. Res. Technol..

[39] Jena KK, Rout TK, Narayan R, Raju KV (2012). Novel organic-inorganic hybrid coatings prepared by the sol-gel process: Corrosion and mechanical properties. Polym. Int..

[40] Gončuková Z, Řezanka M, Dolina J, Dvořák L (2021). Sulfonated polyethersulfone membrane doped with ZnO-APTES nanoparticles with antimicrobial properties. React. Funct. Polym..

[41] Sardarian AR, Zangiabadi M, Inaloo DI (2016). Fe_3_O_4_@SiO_2_/Schiff base/Pd complex as an efficient heterogeneous and recyclable nanocatalyst for chemoselective *N*-arylation of *O*-alkyl primary carbamates. RSC Adv..

[42] Sardarian AR, Inaloo DI, Zangiabadi M (2019). An Fe_3_O_4_@SiO_2_/Schiff base/Cu(II) complex as an efficient recyclable magnetic nanocatalyst for selective mono *N*-arylation of primary *O*-alkyl thiocarbamates and primary *O*-alkyl carbamates with aryl halides and arylboronic acids. New J. Chem..

[43] Inaloo DI, Majnooni S (2019). A Fe_3_O_4_@SiO_2_/Schiff base/Pd complex as an efficient heterogeneous and recyclable nanocatalyst for one-pot domino synthesis of carbamates and unsymmetrical ureas. Eur. J. Org. Chem..

[44] Li T, Pang H, Wu Q, Huang M, Xu J, Zheng L, Wang B, Qiao Y (2022). Rigid Schiff base complex supermolecular aggregates as a high-performance pH probe: Study on the enhancement of the aggregation-caused quenching (ACQ) effect via the substitution of halogen atoms. Int. J. Mol. Sci..

[45] Wasly H, Abd El-Sadek M, Henini M (2018). Influence of reaction time and synthesis temperature on the physical properties of ZnO nanoparticles synthesized by the hydrothermal method. Appl. Phys. A.

[46] Mahdavi R, Talesh SSA (2022). Effects of amine (APTES) and thiol (MPTMS) silanes-functionalized ZnO NPs on the structural, morphological and selective sonophotocatalysis of mixed pollutants: Box-Behnken design (BBD). J. Alloys Compd..

[47] Qi X, Yoon H, Lee S-H, Yoon J, Kim S-J (2008). Surface-modified imogolite by 3-APS-OsO_4_ complex: Synthesis, characterization and its application in the dihydroxylation of olefins. J. Ind. Eng. Chem..

[48] Nath MR, Ahmed AN, Gafur MA, Miah MY, Bhattacharjee S (2018). ZnO nanoparticles preparation from spent zinc-carbon dry cell batteries: studies on structural, morphological and optical properties. J. Asian Ceram. Soc..

[49] Khan M, Khan M, Kuniyil M, Adil SF, Al-Warthan A, Alkhathlan HZ, Tremel W, Tahir MN, Siddiqui MRH (2014). Biogenic synthesis of palladium nanoparticles using Pulicaria glutinosa extract and their catalytic activity towards the Suzuki coupling reaction. Dalton Trans..

[50] Zeng M, Wang Y, Liu Q, Yuan X, Feng R, Yang Z, Qi C (2016). N-doped mesoporous carbons supported palladium catalysts prepared from chitosan/silica/palladium gel beads. Int. J. Biol. Macromol..

[51] Xu Q, Cai M (2007). MCM-41-supported poly(γ-mercaptopropylsiloxane palladium(0)) complex as an efficient catalyst for Heck arylation of conjugated alkenes with aryl halides. React. Funct. Polym..

[52] Huang X-J, Dong F, Chen L, Li Y-Q (2008). Nanopalladium immobilized on aminoethanol-functionalized poly (vinyl chloride): An easily prepared, air and moisture stable catalyst for Heck reactions. Monatsh. Chem..

[53] Cai M, Huang Y, Zhao H, Song C (2017). Silica-supported bidentate arsine palladium(0) complex: A highly active and stereoselective catalyst for arylation of conjugated alkenes. J. Organomet. Chem..

[54] Nie G, Zhang L, Cui Y (2012). Synthesis of polyphenol microsphere-supported palladium complex and evaluation of its catalytic performance for Heck reaction. Appl. Organomet. Chem..

[55] Shendage SS, Nagarkar JM (2014). Electrochemically codeposited reduced graphene oxide and palladium nanoparticles: An efficient heterogeneous catalyst for Heck coupling reaction. Colloids Interface Sci. Commun..

[56] Ohtaka A, Tamaki Y, Igawa Y, Egami K, Shimomura O, Nomura R (2010). Polyion complex stabilized palladium nanoparticles for Suzuki and Heck reaction in water. Tetrahedron.

[57] Tahmasbi B, Ghorbani-Choghamarani A (2017). Pd(0)-Arg-boehmite: As reusable and efficient nanocatalyst in Suzuki and Heck reactions. Catal. Lett..

[58] Keesara S, Parvathaneni S, Dussa G, Mandapati MR (2014). Polystyrene supported thiopseudourea Pd(II) complex: Applications for Sonogashira, Suzuki-Miyaura, Heck, Hiyama and Larock heteroannulation reactions. J. Organomet. Chem..

[59] Tamami BH, Ghasemi AS, Farjadian F (2011). Palladium nanoparticles supported on poly(*N*-vinylpyrrolidone)-grafted silica as new recyclable catalyst for Heck cross-coupling reactions. J. Organomet. Chem..

[60] Jaleh B, Mousavi SS, Sajjadi M, Eslamipanah M, Maryaki JM, Orooji Y, Varma RS (2023). Synthesis of bentonite/Ag nanocomposite by laser ablation in air and its application in remediation. Chemosphere.

[61] Zhu Y, Shen J, Zhou K, Chen C, Yang X, Li C (2011). Multifunctional magnetic composite microspheres with in situ growth Au nanoparticles: A highly efficient catalyst system. J. Phys. Chem. C.

[62] Kuroda K, Ishida T, Haruta M (2009). Reduction of 4-nitrophenol to 4-aminophenol over Au nanoparticles deposited on PMMA. J. Mol. Catal. A Chem..

[63] Kassem AA, Abdelhamid HN, Fouad DM, Ibrahim SA (2021). Catalytic reduction of 4-nitrophenol using copper terephthalate frameworks and CuO@C composite. J. Environ. Chem. Eng..

[64] Ma M, Yang Y, Li W, Feng R, Li Z, Lyu P, Ma Y (2019). Gold nanoparticles supported by amino groups on the surface of magnetite microspheres for the catalytic reduction of 4-nitrophenol. J. Mater. Sci..

[65] Liang M, Su R, Qi W, Yu Y, Wang L, He Z (2014). Synthesis of well-dispersed Ag nanoparticles on eggshell membrane for catalytic reduction of 4-nitrophenol. J. Mater. Sci..

[66] Alshehri SM, Almuqati T, Almuqati N, Al-Farraj E, Alhokbany N, Ahamad T (2016). Chitosan based polymer matrix with silver nanoparticles decorated multiwalled carbon nanotubes for catalytic reduction of 4-nitrophenol. Carbohydr. Polym..

[67] Tian Y, Liu Y, Pang F, Wang F, Zhang X (2015). Green synthesis of nanostructed Ni-reduced graphene oxide hybrids and their application for catalytic reduction of 4-nitrophenol. Colloids Surf. A Physicochem. Eng. Asp..

[68] Zhang P, Yang X, Peng H, Liu D, Lu H, Wei J, Gui J (2017). Magnetically recoverable hierarchical Pt/Fe_2_O_3_ microflower: Superior catalytic activity and stability for reduction of 4-nitrophenol. Catal. Commun..

[69] Das TK, Remanan S, Ghosh S, Das NC (2021). An environment friendly free-standing cellulose membrane derived for catalytic reduction of 4-nitrophenol: A sustainable approach. J. Environ. Chem. Eng..

[70] Wang X, Fu J, Wang M, Wang Y, Chen Z, Zhang J, Chen J, Xu Q (2014). Facile synthesis of Au nanoparticles supported on polyphosphazene functionalized carbon nanotubes for catalytic reduction of 4-nitrophenol. J. Mater. Sci..

[71] Zheng Y, Shu J, Wang Z (2015). AgCl@Ag composites with rough surfaces as bifunctional catalyst for the photooxidation and catalytic reduction of 4-nitrophenol. Mater. Lett..

