# Supplementary material for: Synthesis of palladium nanoparticles stabilized on Schiff base-modified ZnO particles as a nanoscale catalyst for the phosphine-free Heck coupling reaction and 4-nitrophenol reduction
Source: Sci Rep. 2023 Jul 25;13:12008. doi: 10.1038/s41598-023-38898-w (PMC10368721; doi:10.1038/s41598-023-38898-w)
Supplement: Supplementary file 1 — Supplementary Figures. [file 41598_2023_38898_MOESM1_ESM.docx]

**SUPPLEMENTARY DATA**

**Synthesis of palladium nanoparticles stabilized on Schiff base modified ZnO particles as a nanoscale catalyst for the phosphine-free Heck coupling reaction and 4-nitrophenol reduction**

Nuray Yılmaz Baran^a^, Talat Baran^b^ and Mahmoud Nasrollahzadeh^c^[[1]](#footnote-1)^*^

*^a^Technical Vocational School, Department of Chemistry Technology, Aksaray University, 68100 Aksaray, Turkey*

*^b^Department of Chemistry, Faculty of Science and Letters, Aksaray University, 68100 Aksaray, Turkey*

*^c^Department of Chemistry, Faculty of Science, University of Qom, PO Box 37185‑359, Qom, Iran*


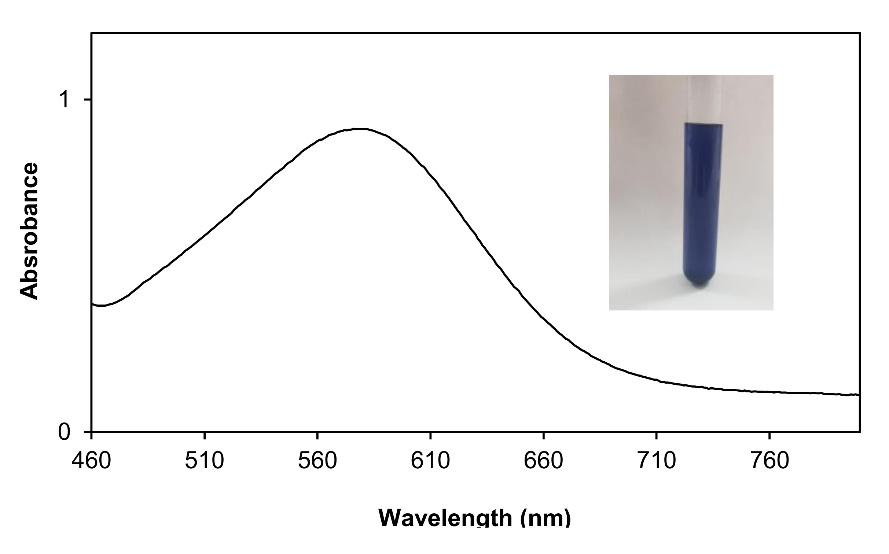


**Figure S1.** Ninhydrin color test of ZnO-NH_2_.


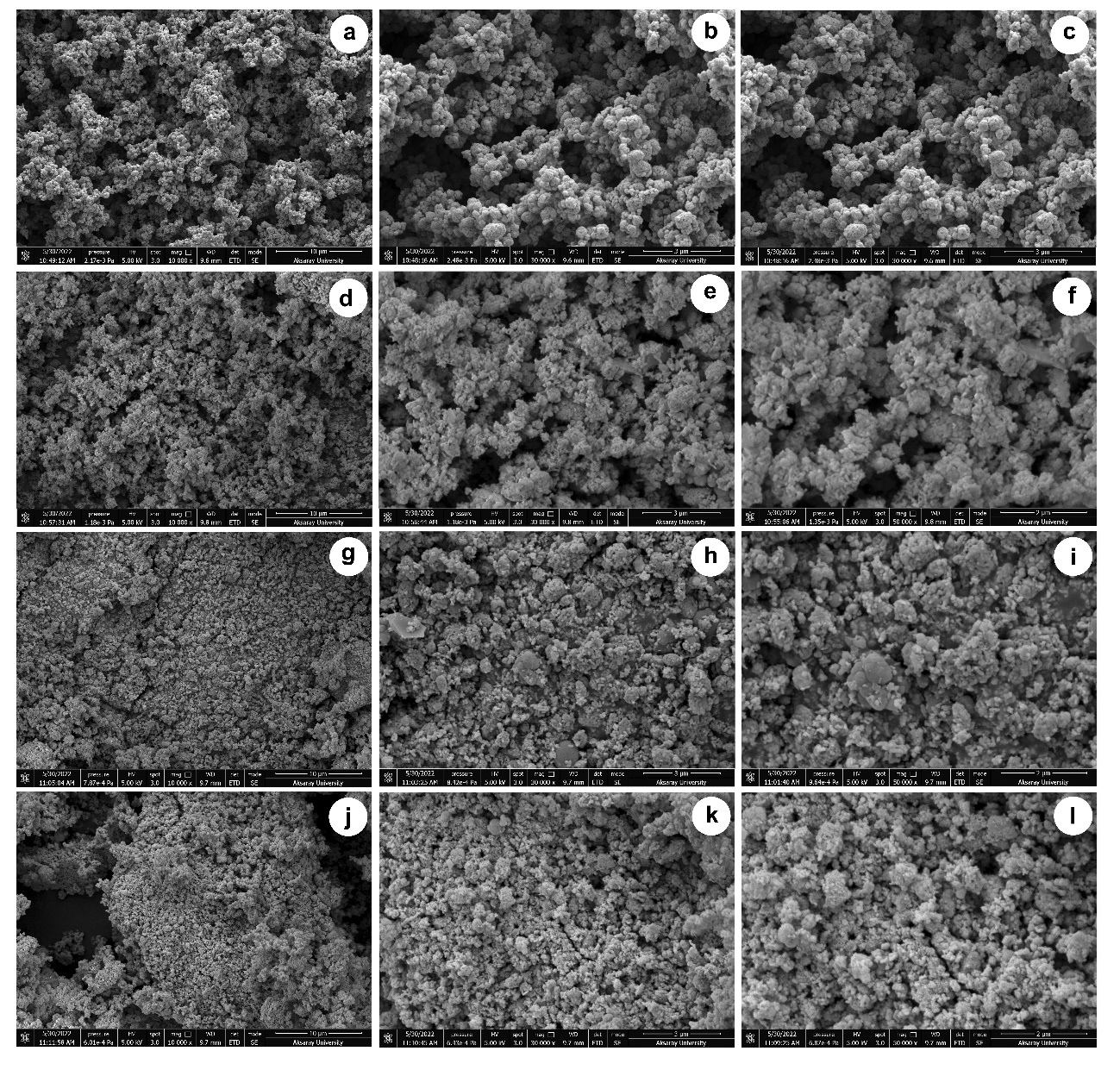


**Figure S2.** FE-SEM micrographs of ZnO (a-c), ZnO-NH_2_ (d-f), ZnO-Scb (g-i) and Pd-ZnO-Scb (j-l).

**
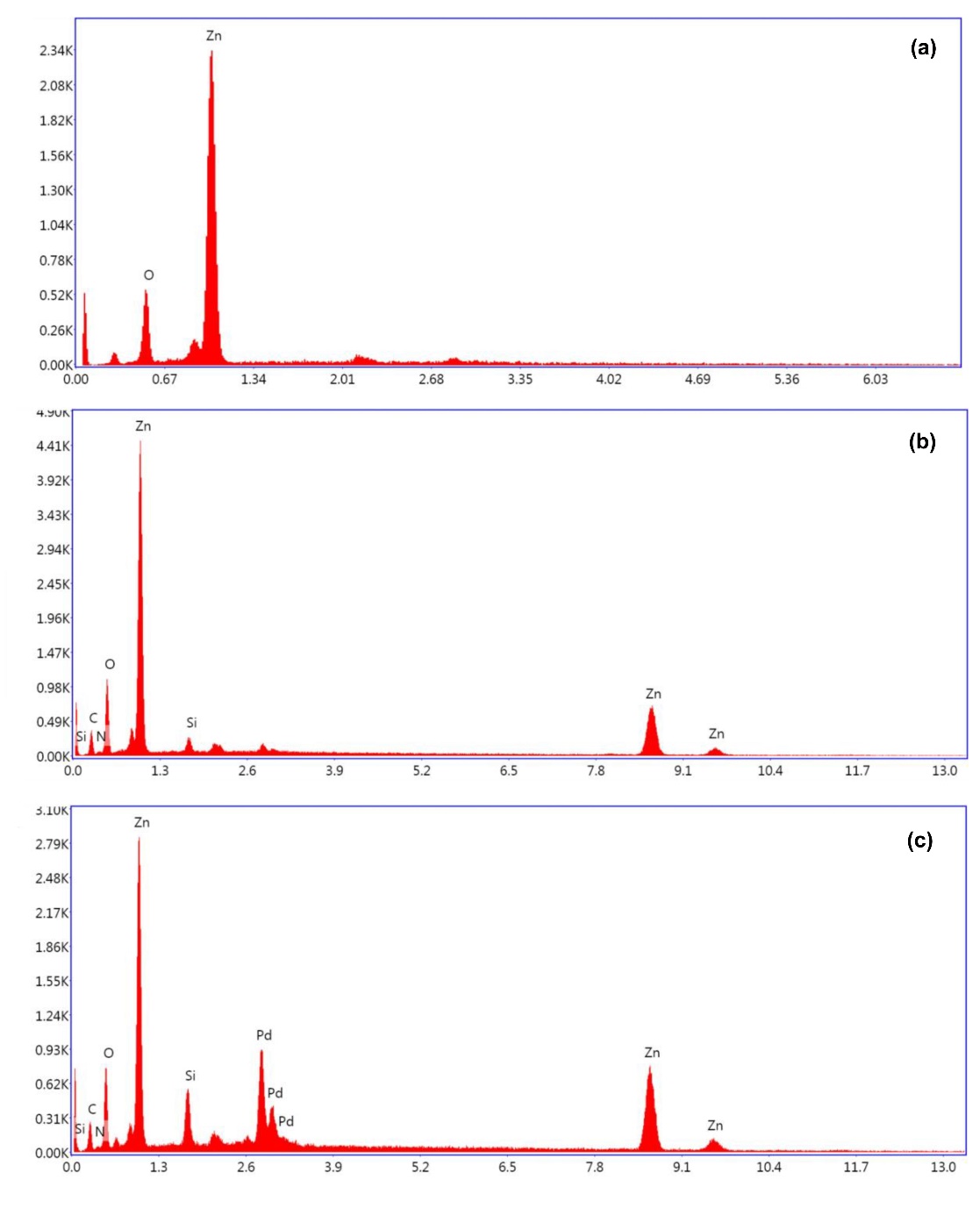
**

**Figure S3.** EDS spectra of ZnO (a), ZnO-NH_2_ (b) and Pd-ZnO-Scb (c).


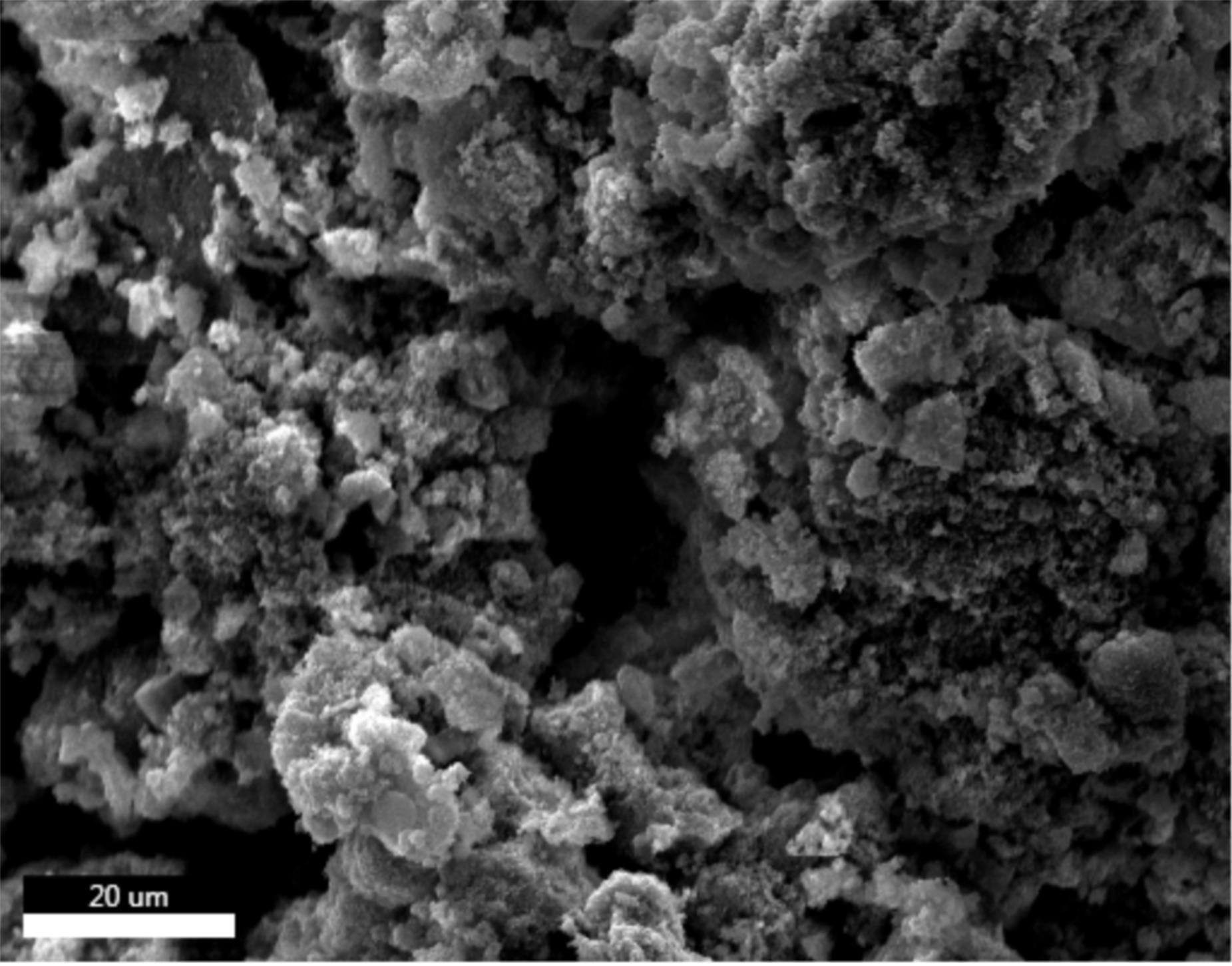

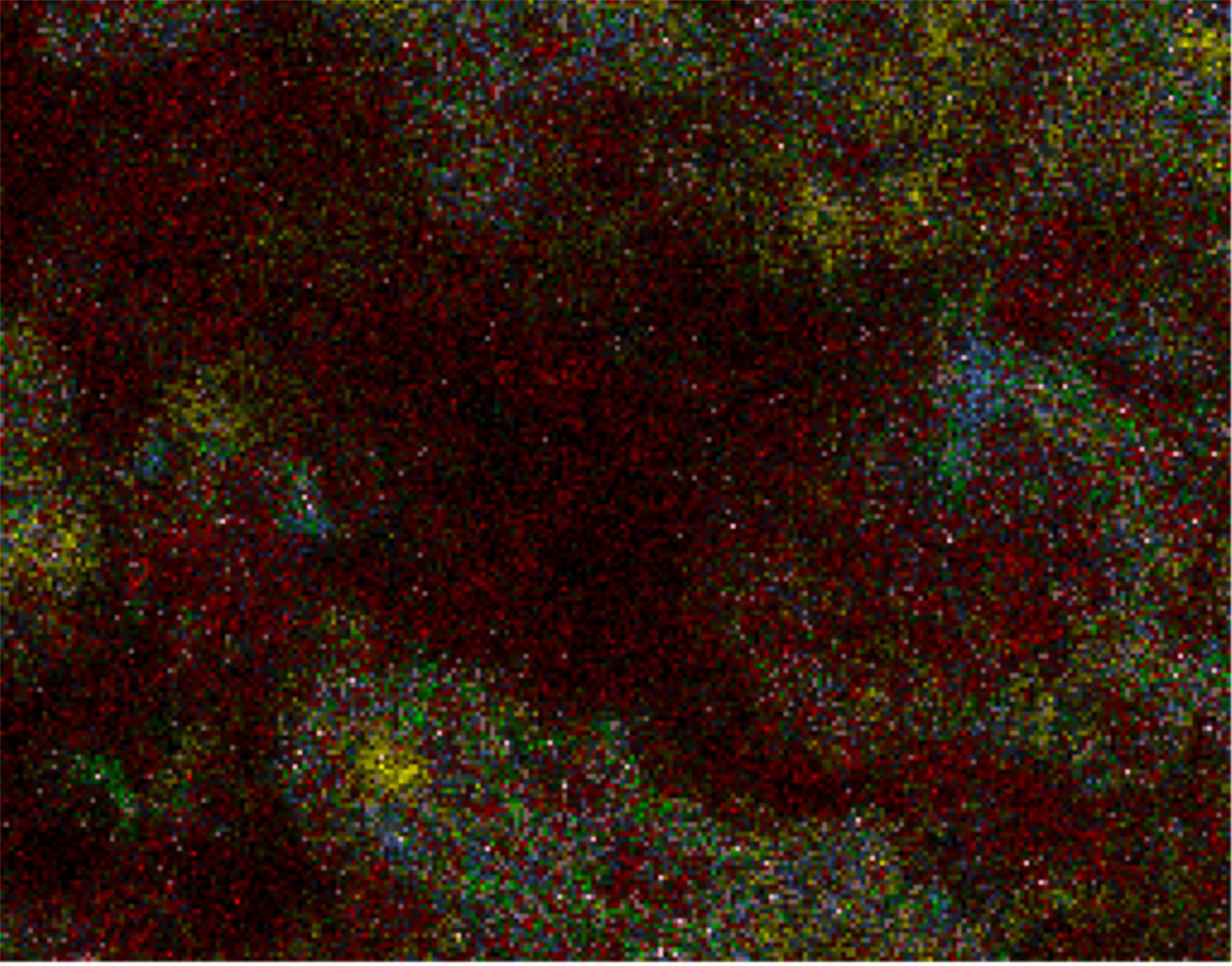


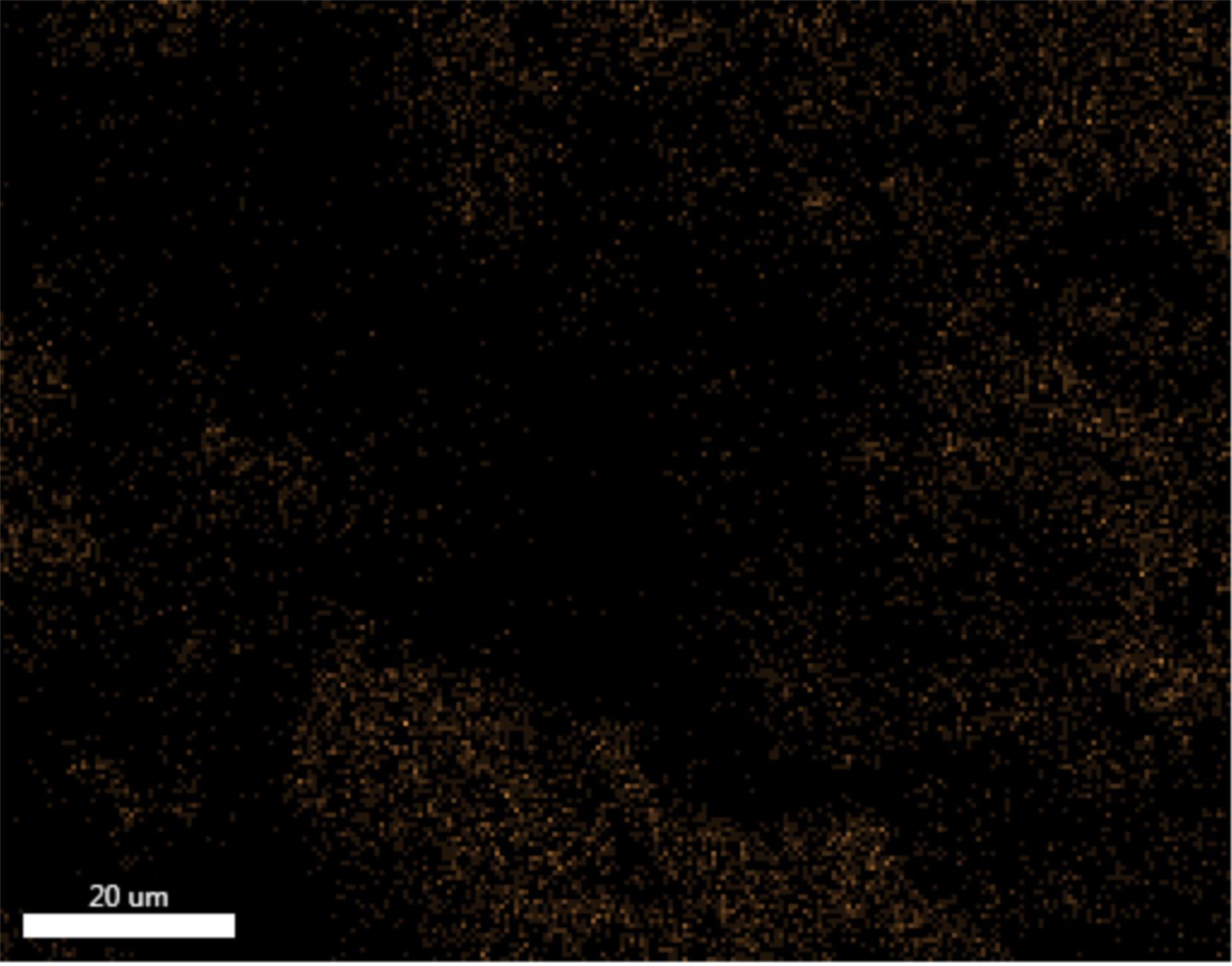

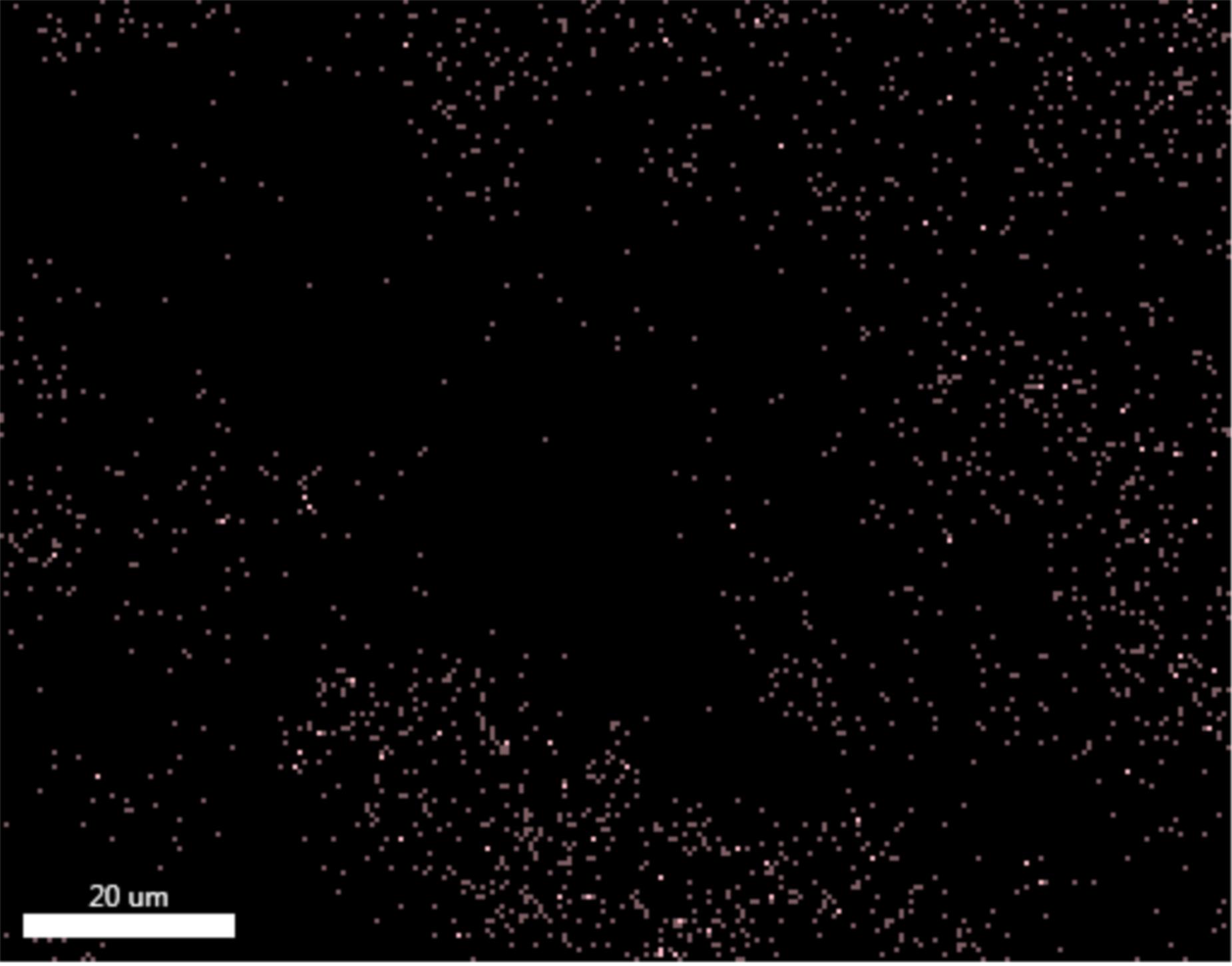


C K_ROI (6) N K_ROI (2)


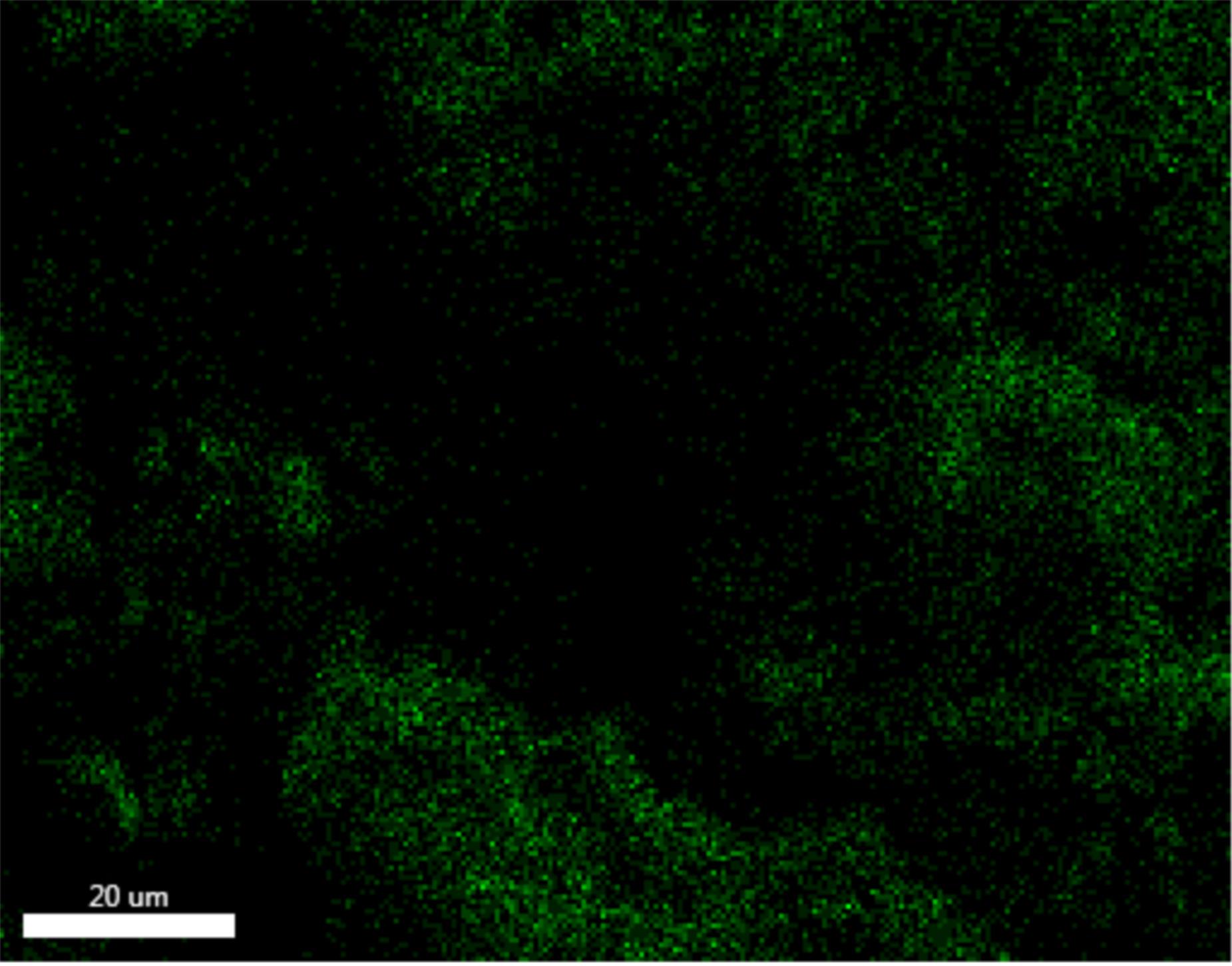

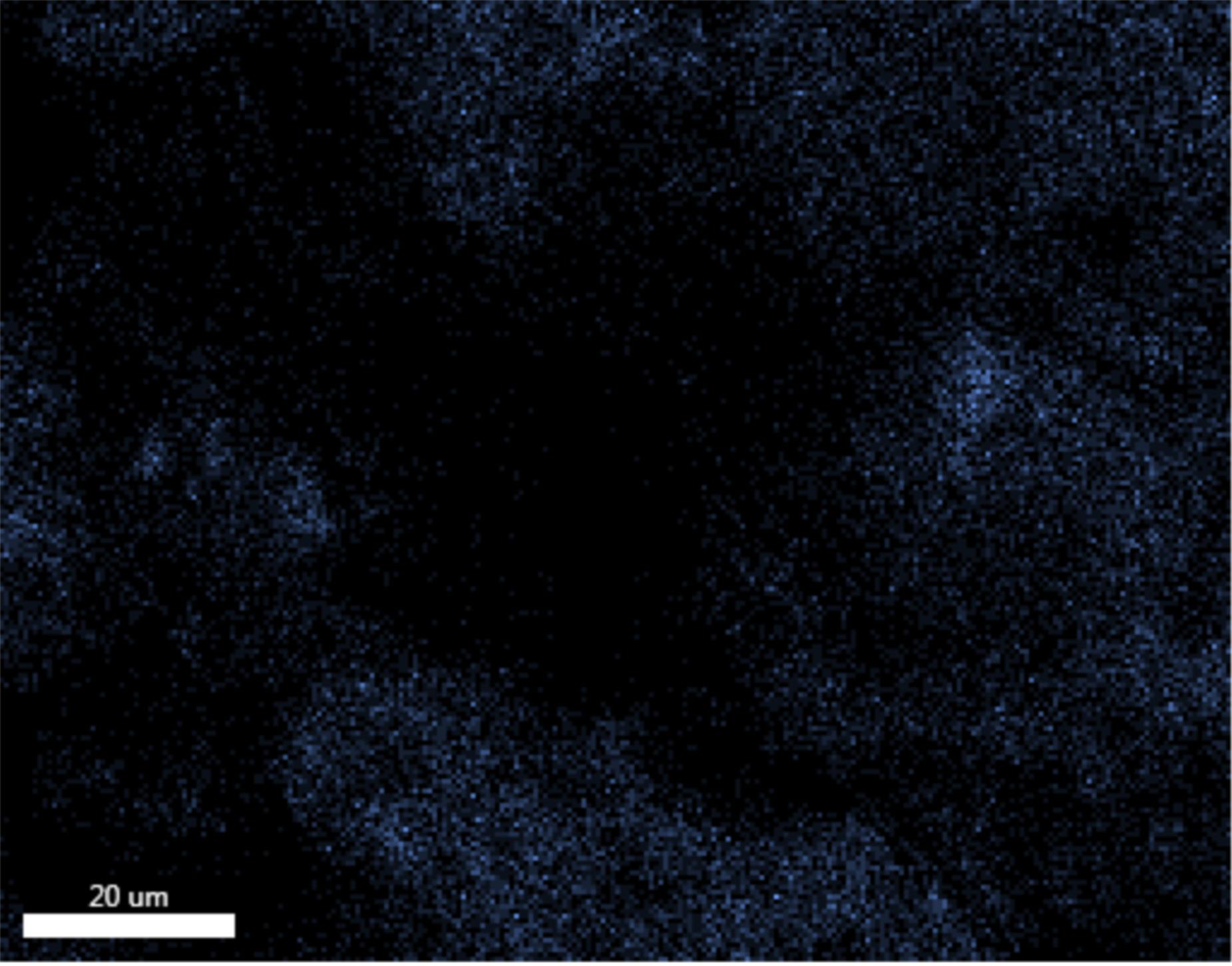


O K_ROI (8) SiK_ROI (9)


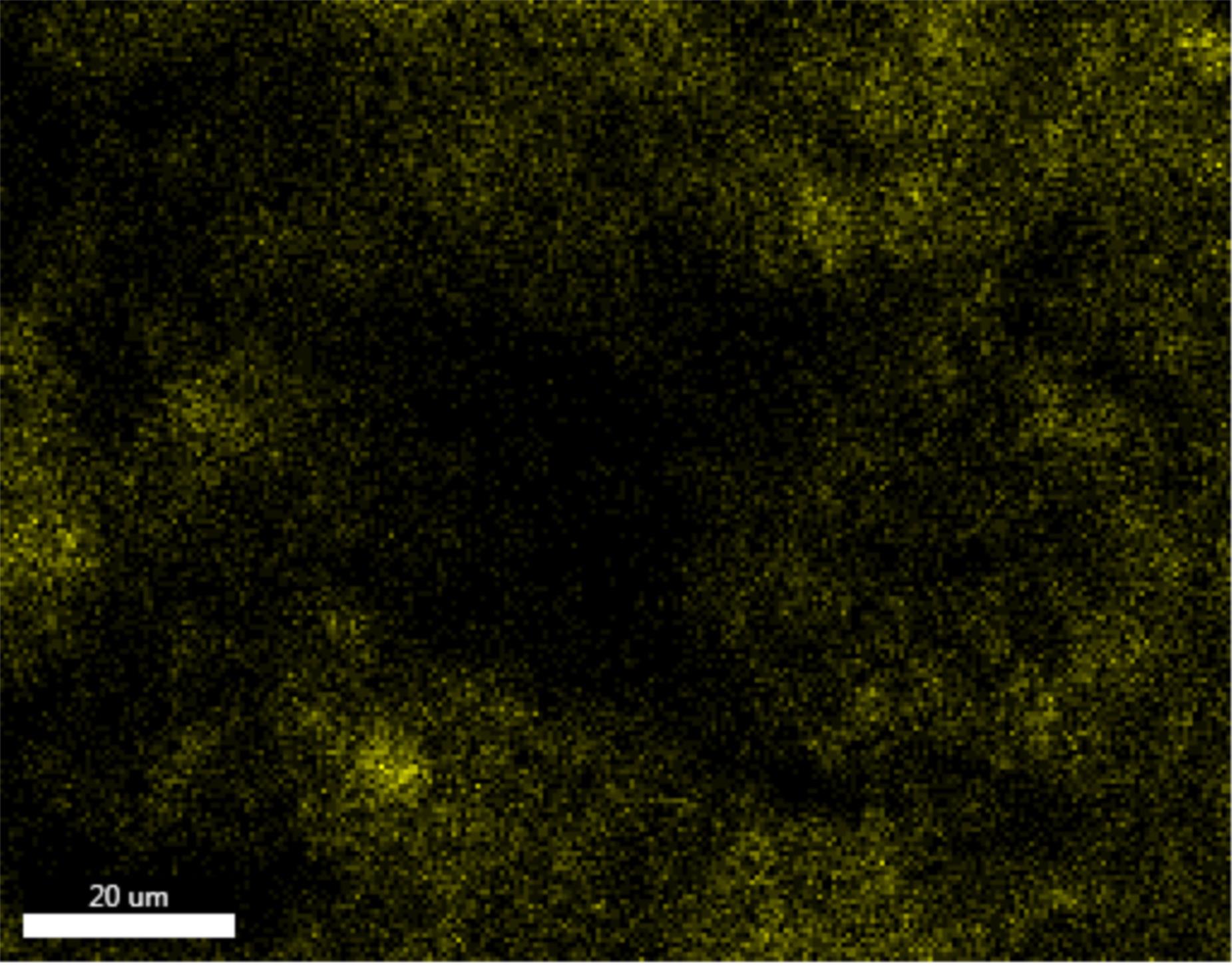

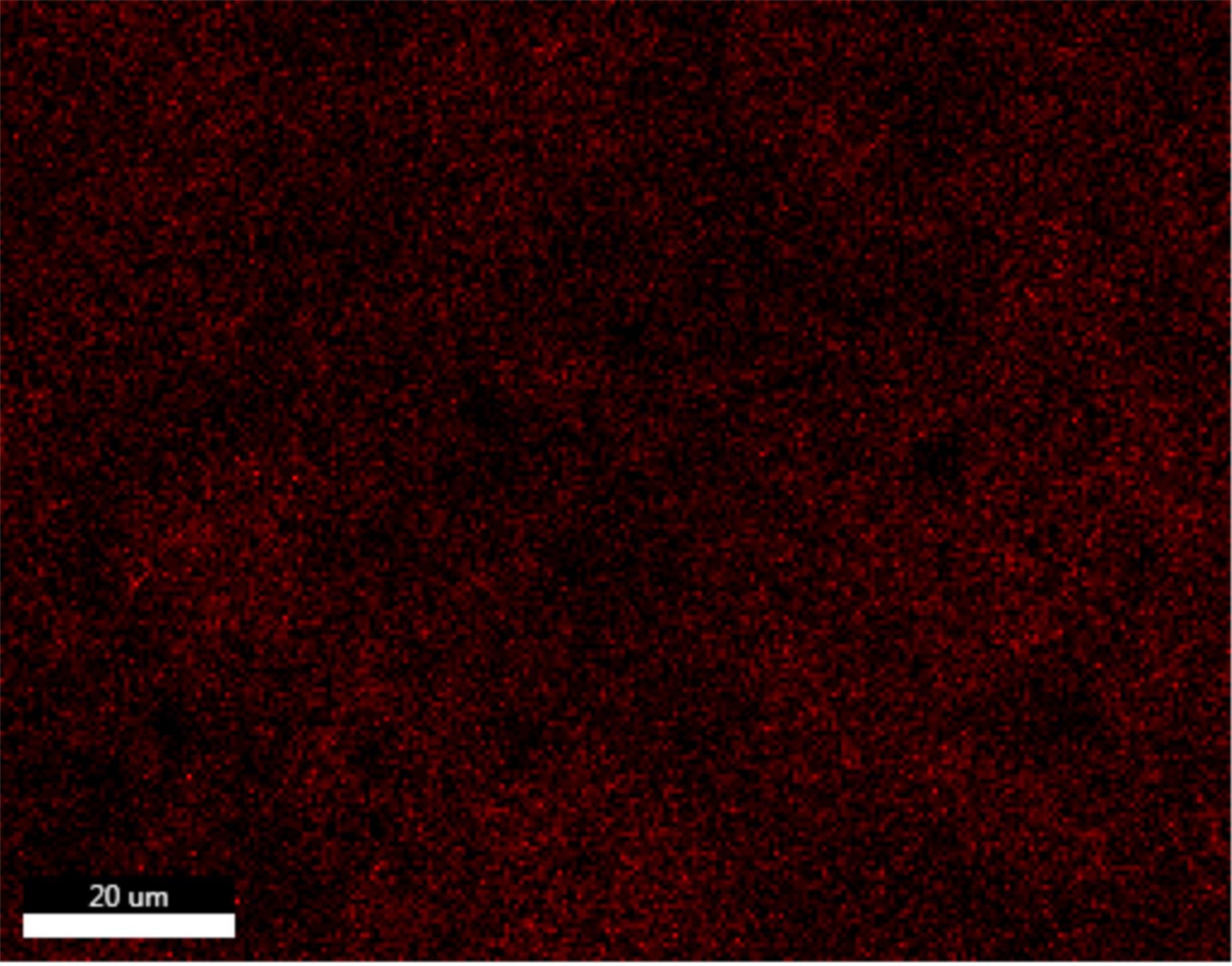


PdL_ROI (15) ZnK_ROI (11)

**Figure S4.** Elemental mapping of Pd-ZnO-Scb.


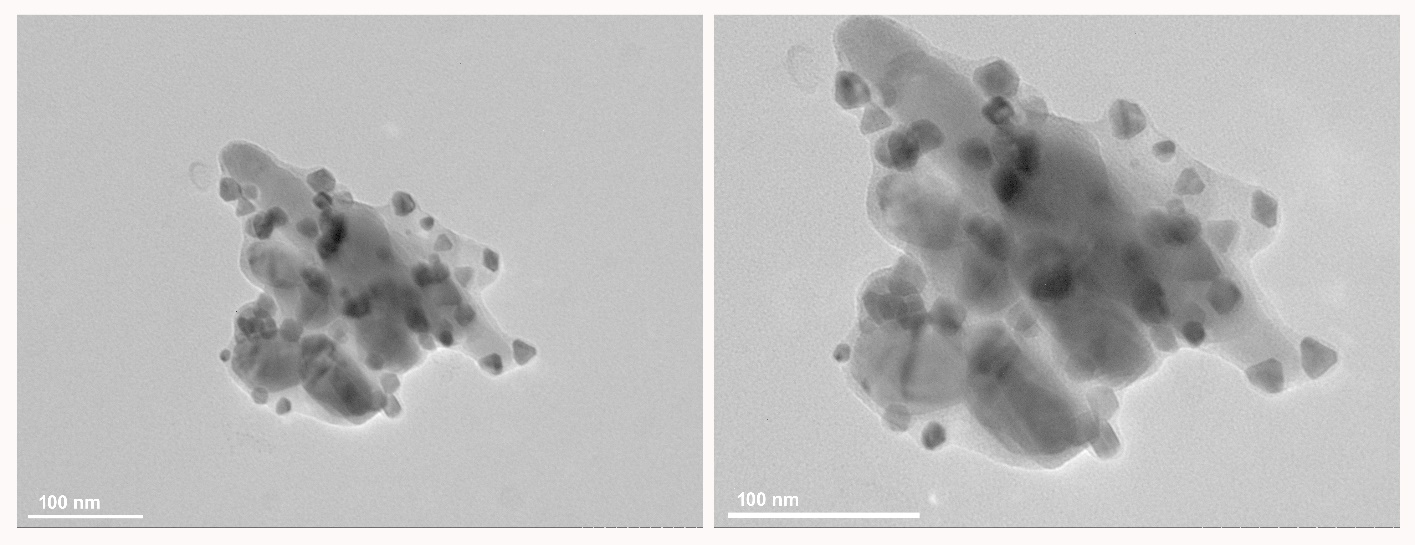


**Figure S5.** TEM images of Pd-ZnO-Scb after seven cycles.


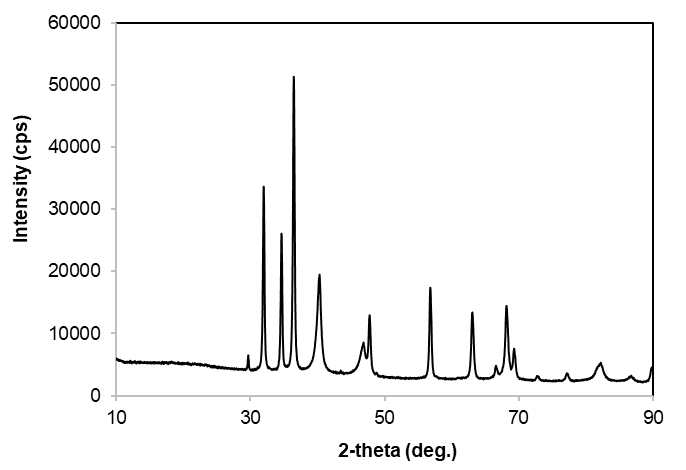


**Figure S6.** XRD pattern of Pd-ZnO-Scb after seven cycles.

**
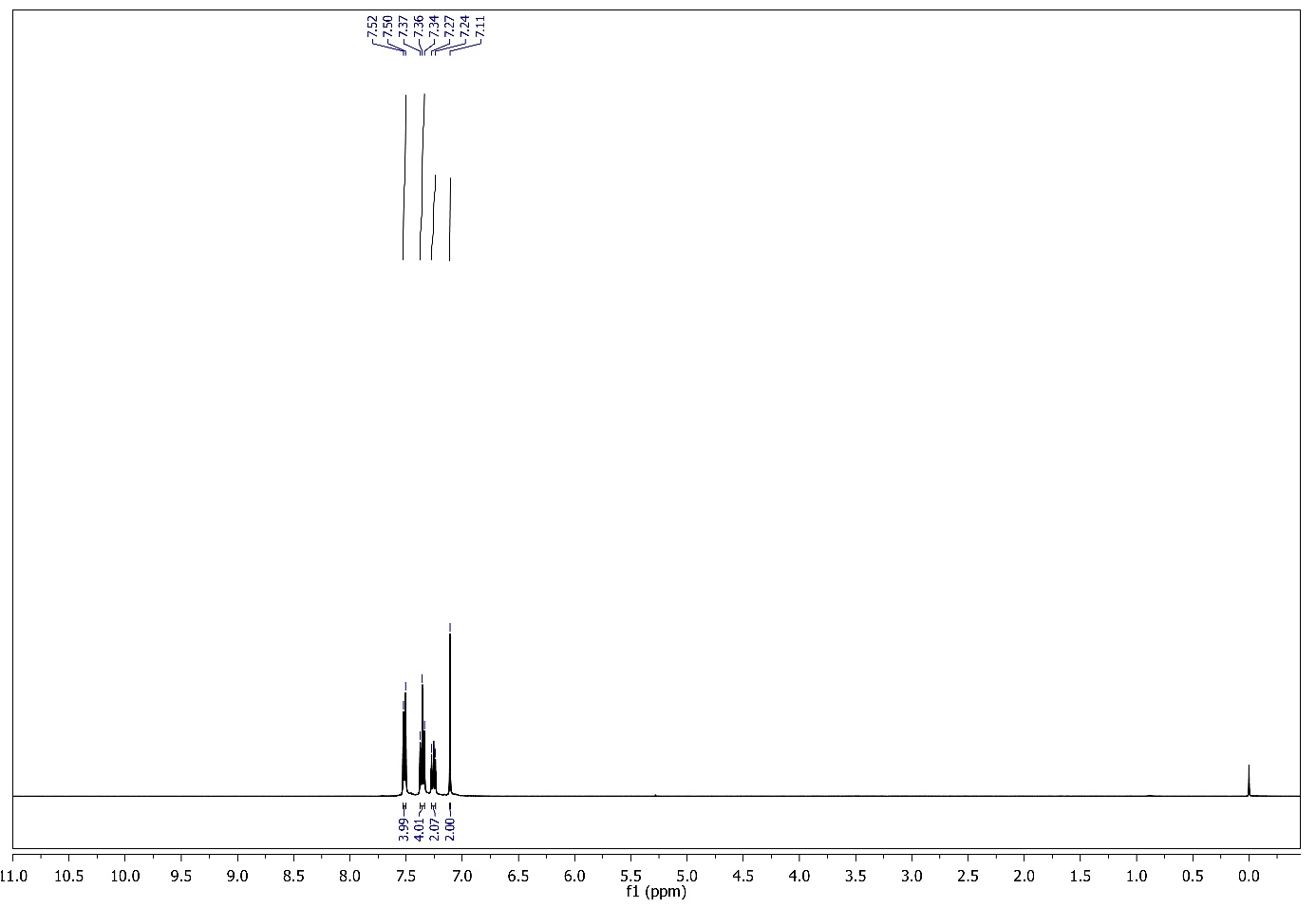
**

**Figure S7.** ^1^H NMR of (E)-1,2-diphenylethene.


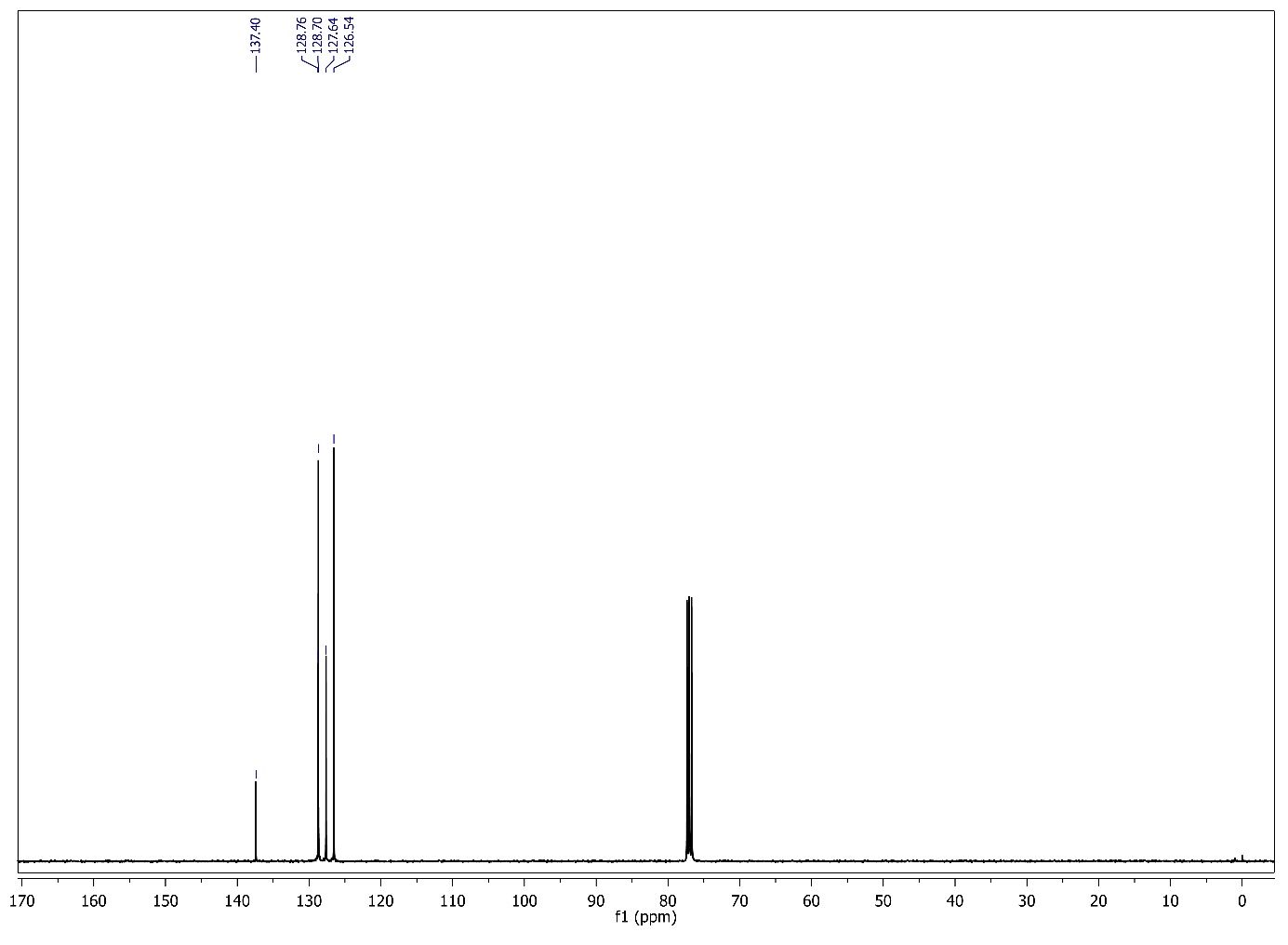


**Figure S8.** ^13^C NMR of (E)-1,2-diphenylethene.

**
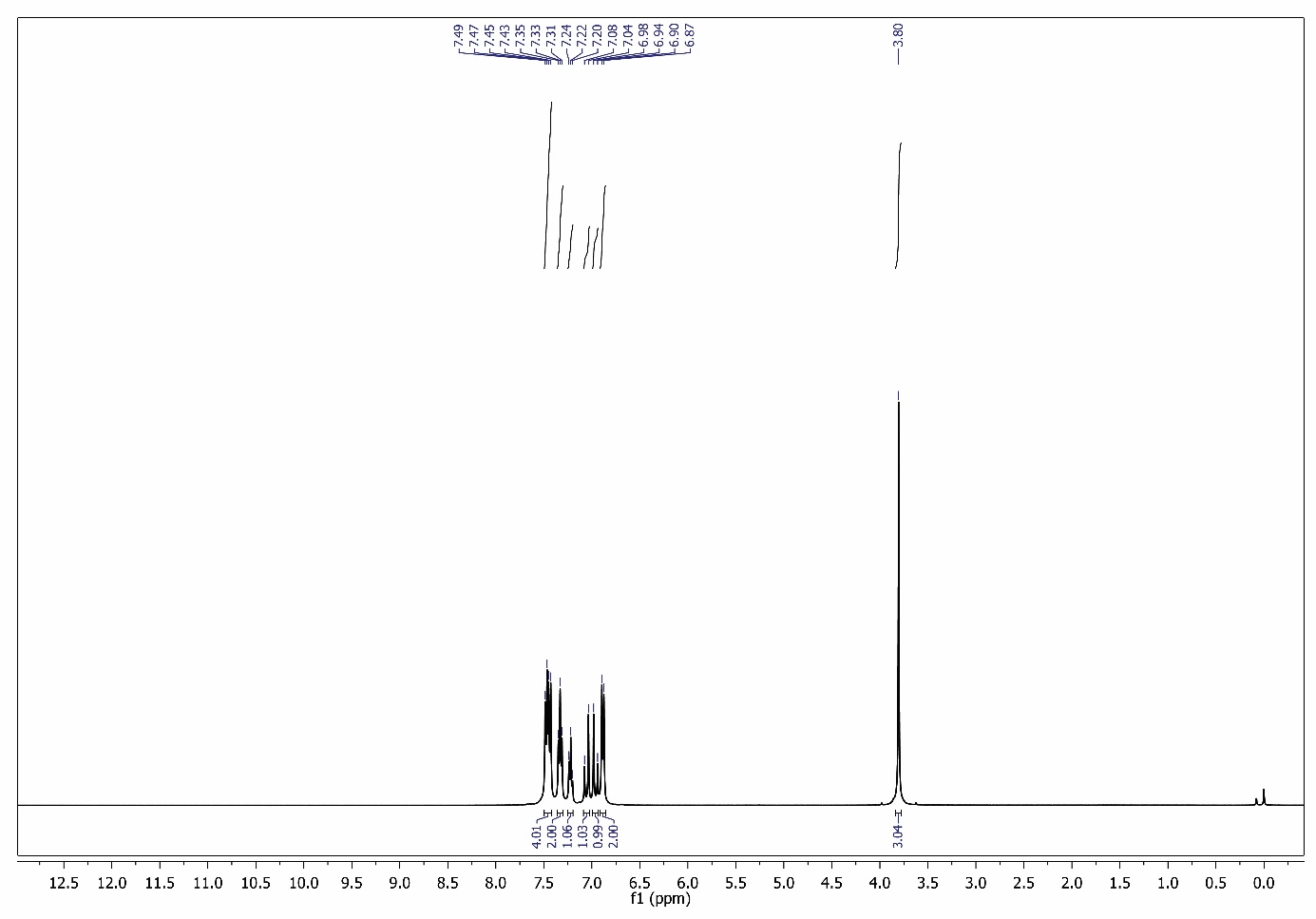
**

**Figure S9.** ^1^H NMR of (E)-1-methoxy-4-styrylbenzene.

*
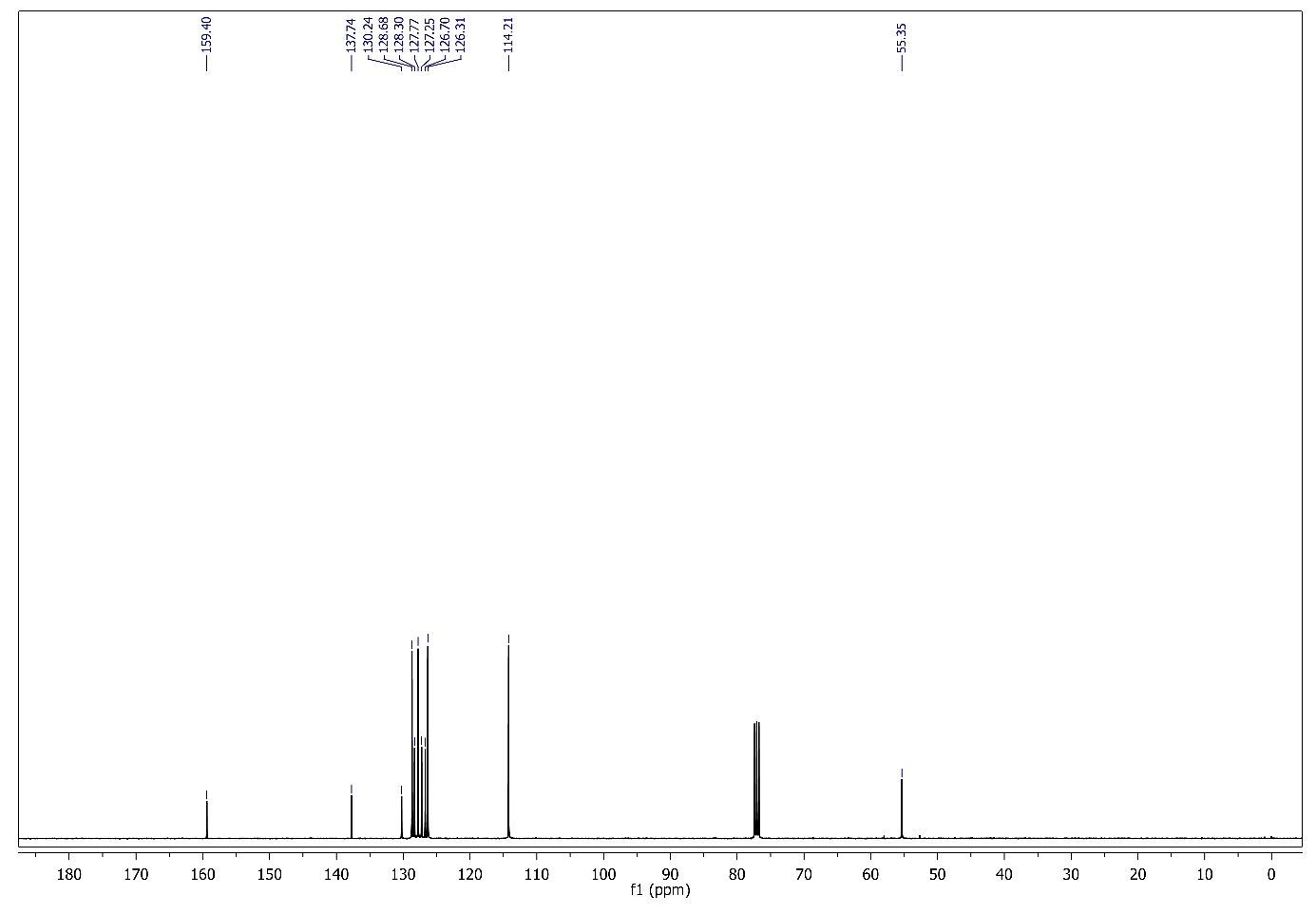
*

**Figure S10.** ^13^C NMR of (E)-1-methoxy-4-styrylbenzene.

**
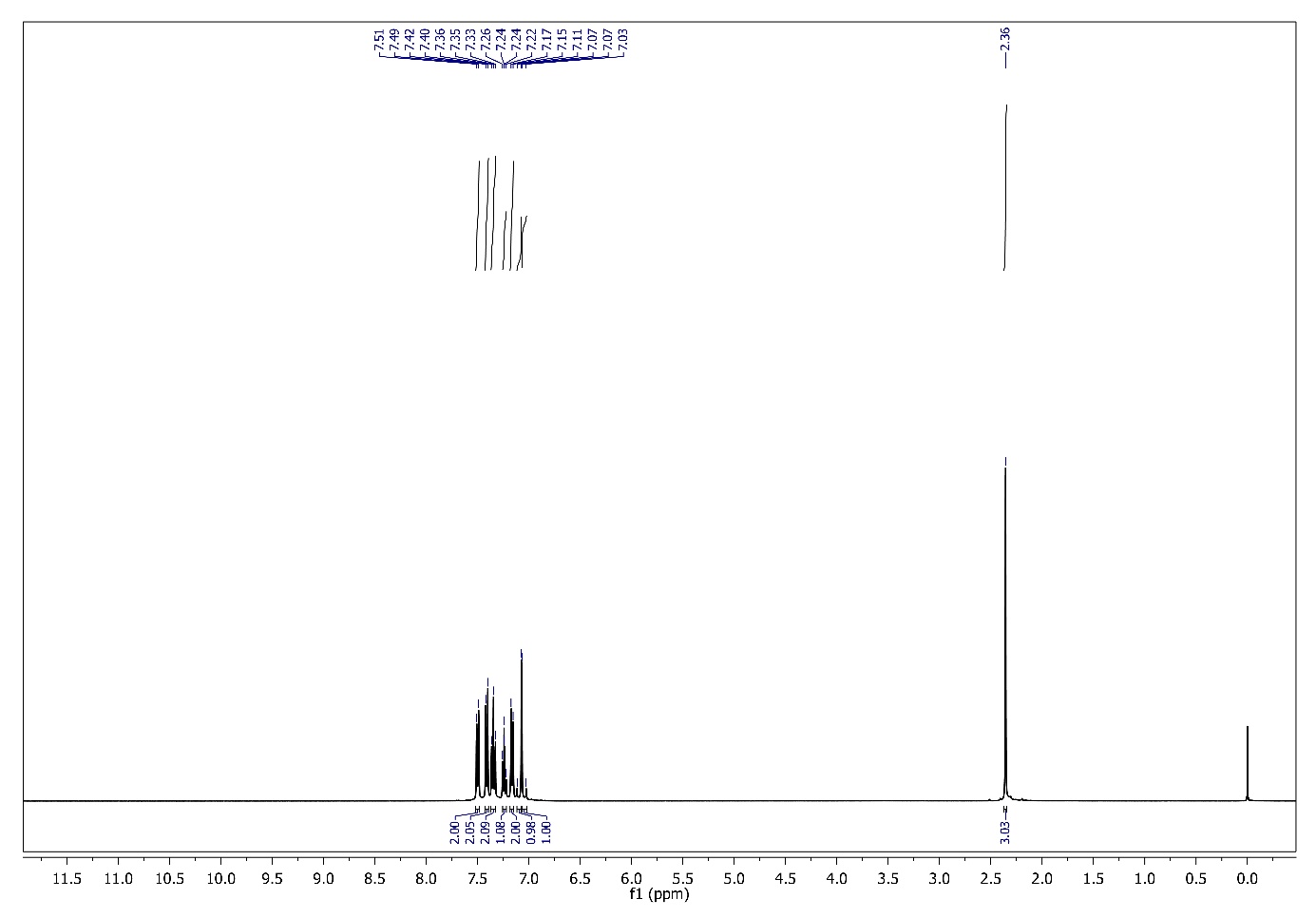
**

**Figure S11.** ^1^H NMR of (E)-1-methyl-4-styrylbenzene.


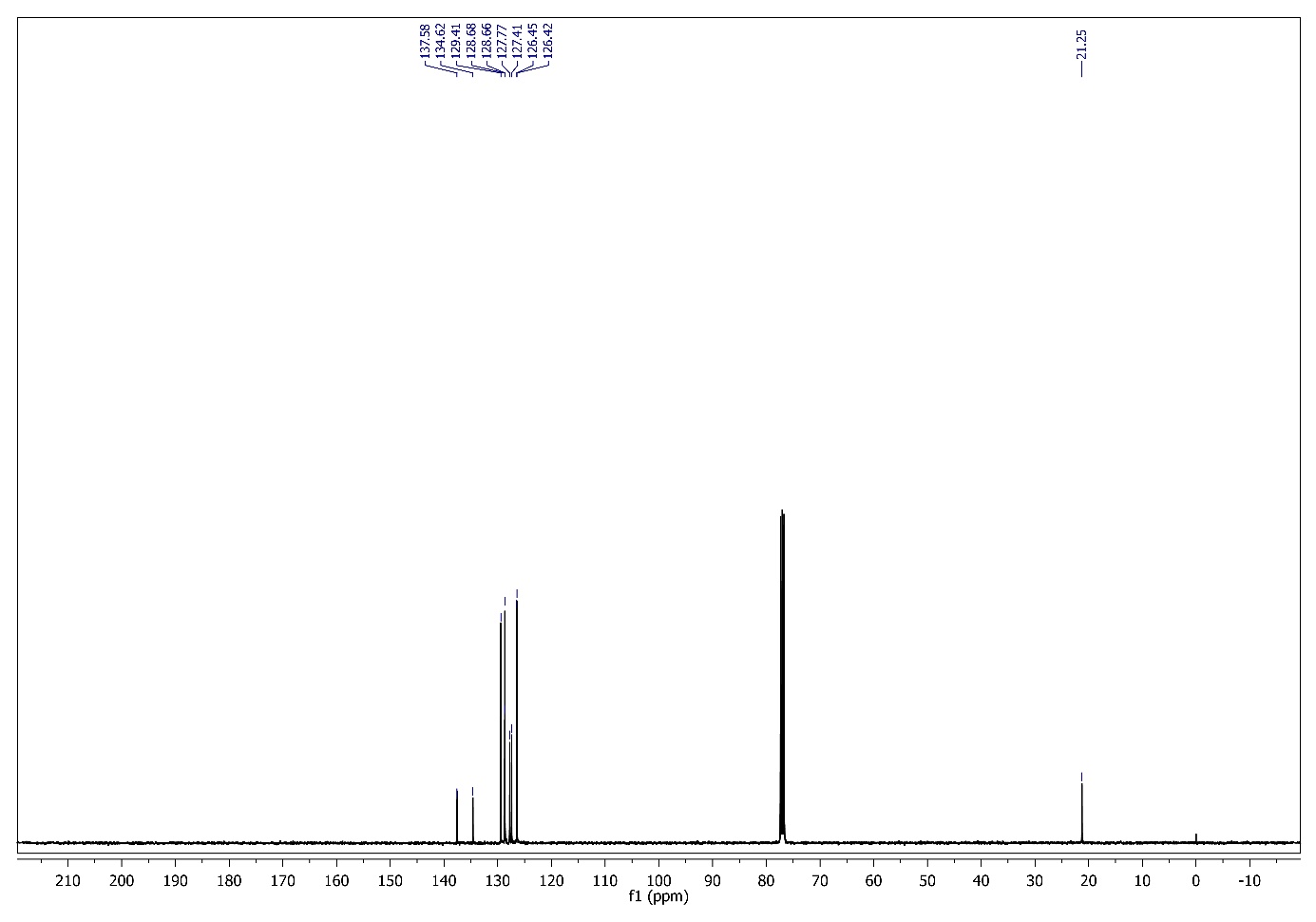


**Figure S12.** ^13^C NMR of (E)-1-methyl-4-styrylbenzene.

**
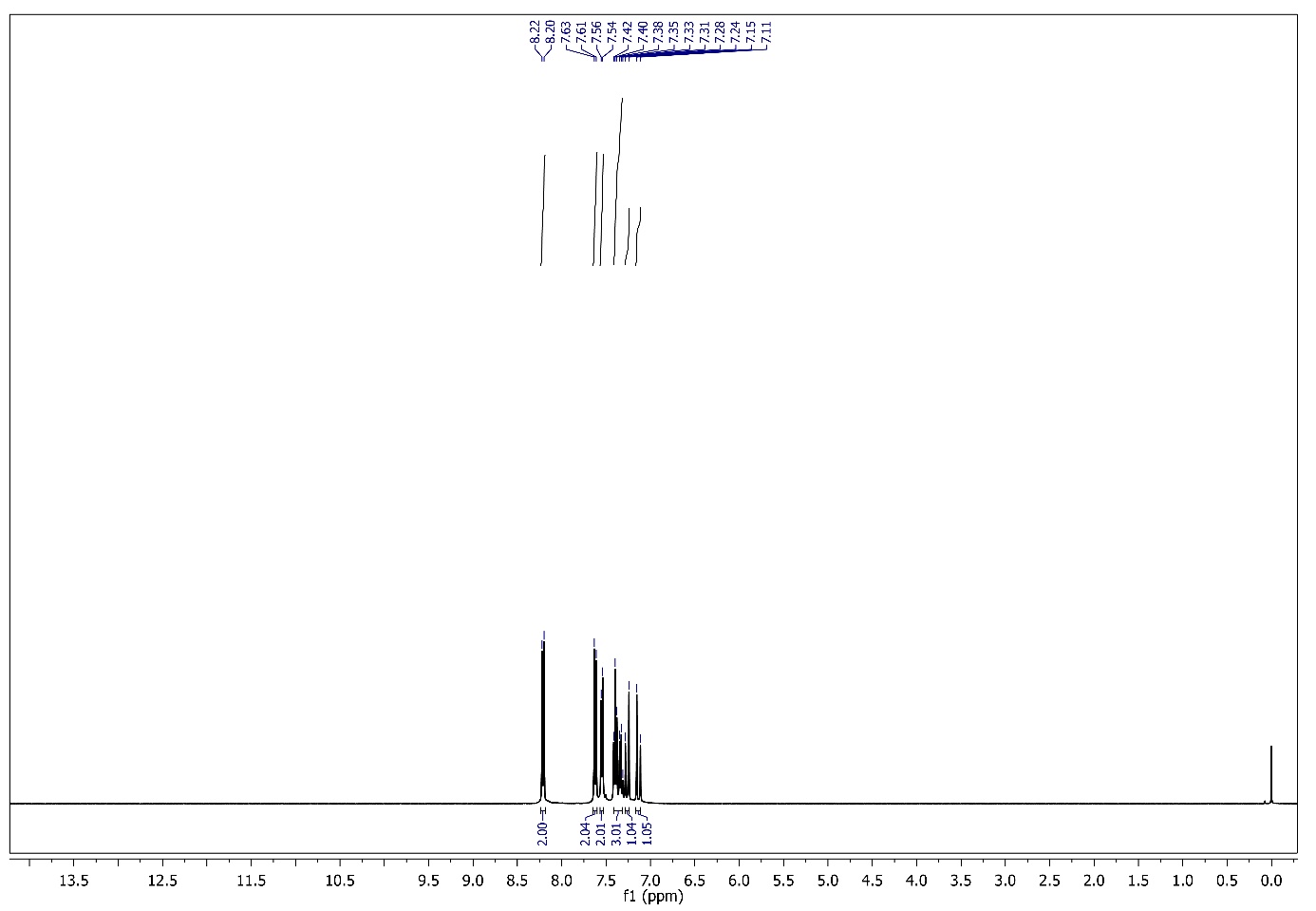
**

**Figure S13.** ^1^H NMR of (E)-1-nitro-4-styrylbenzene.


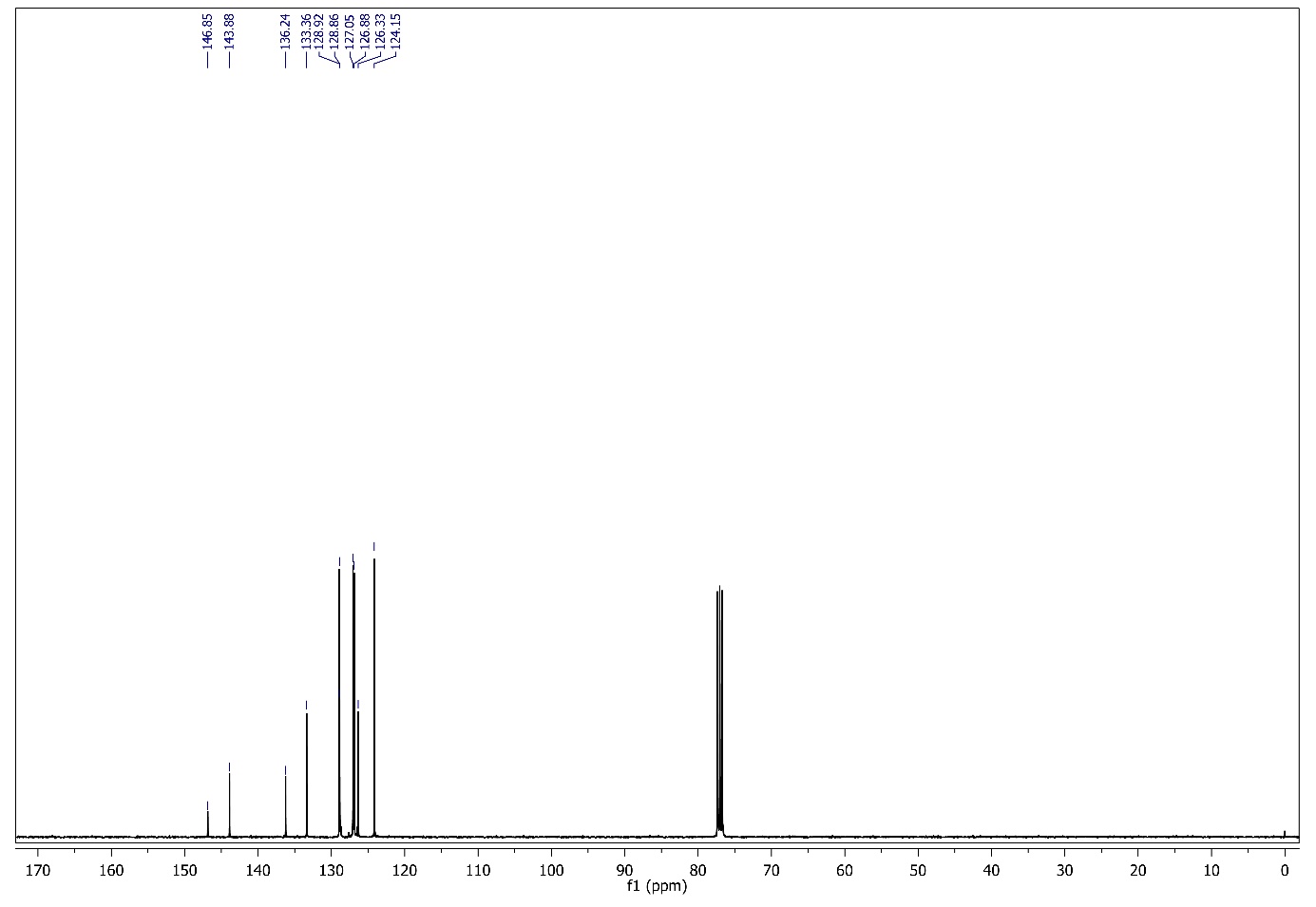


**Figure S14.** ^13^C NMR of (E)-1-nitro-4-styrylbenzene.

**
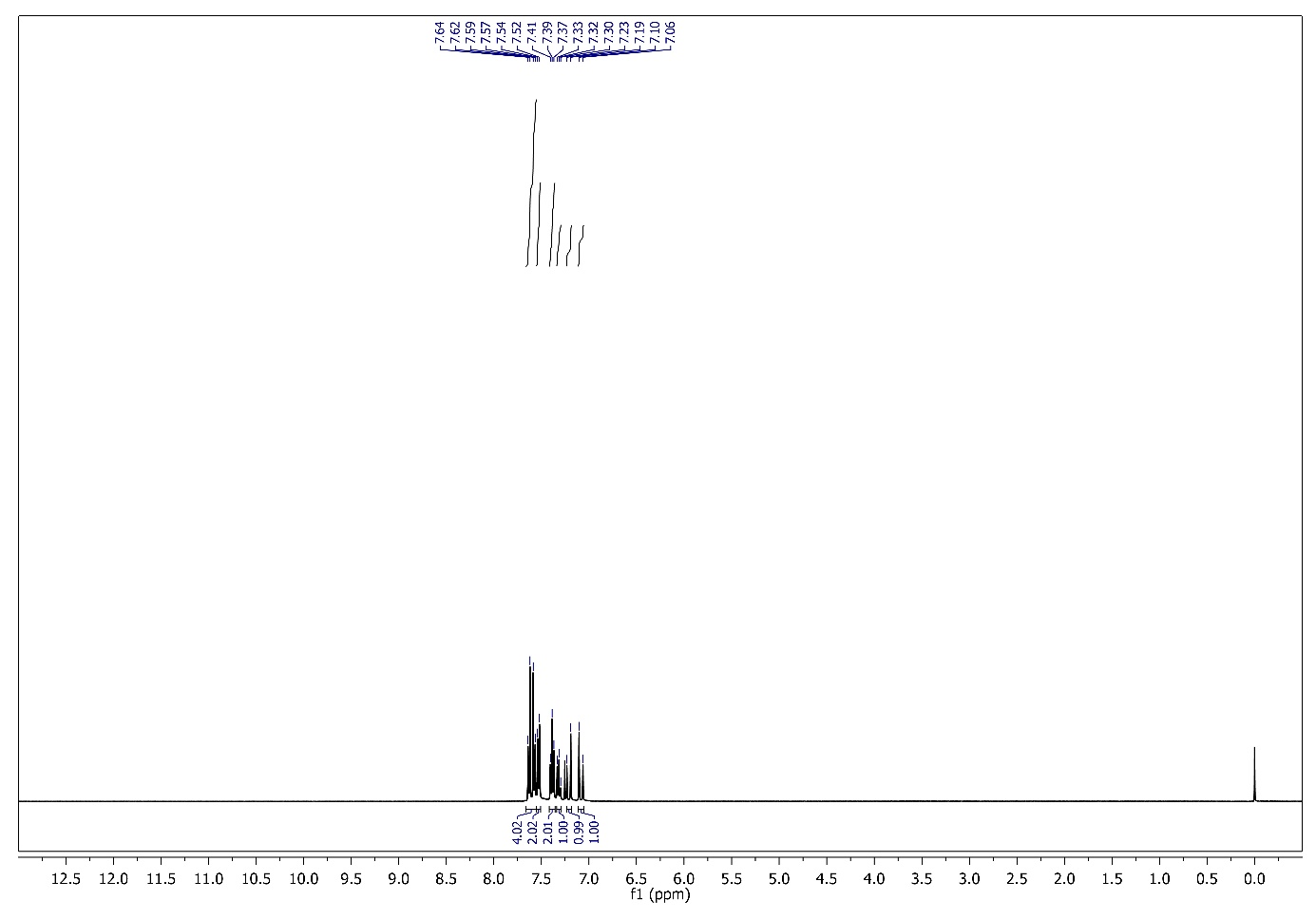
**

**Figure S15.** ^1^H NMR of (E)-4-styrylbenzonitrile.


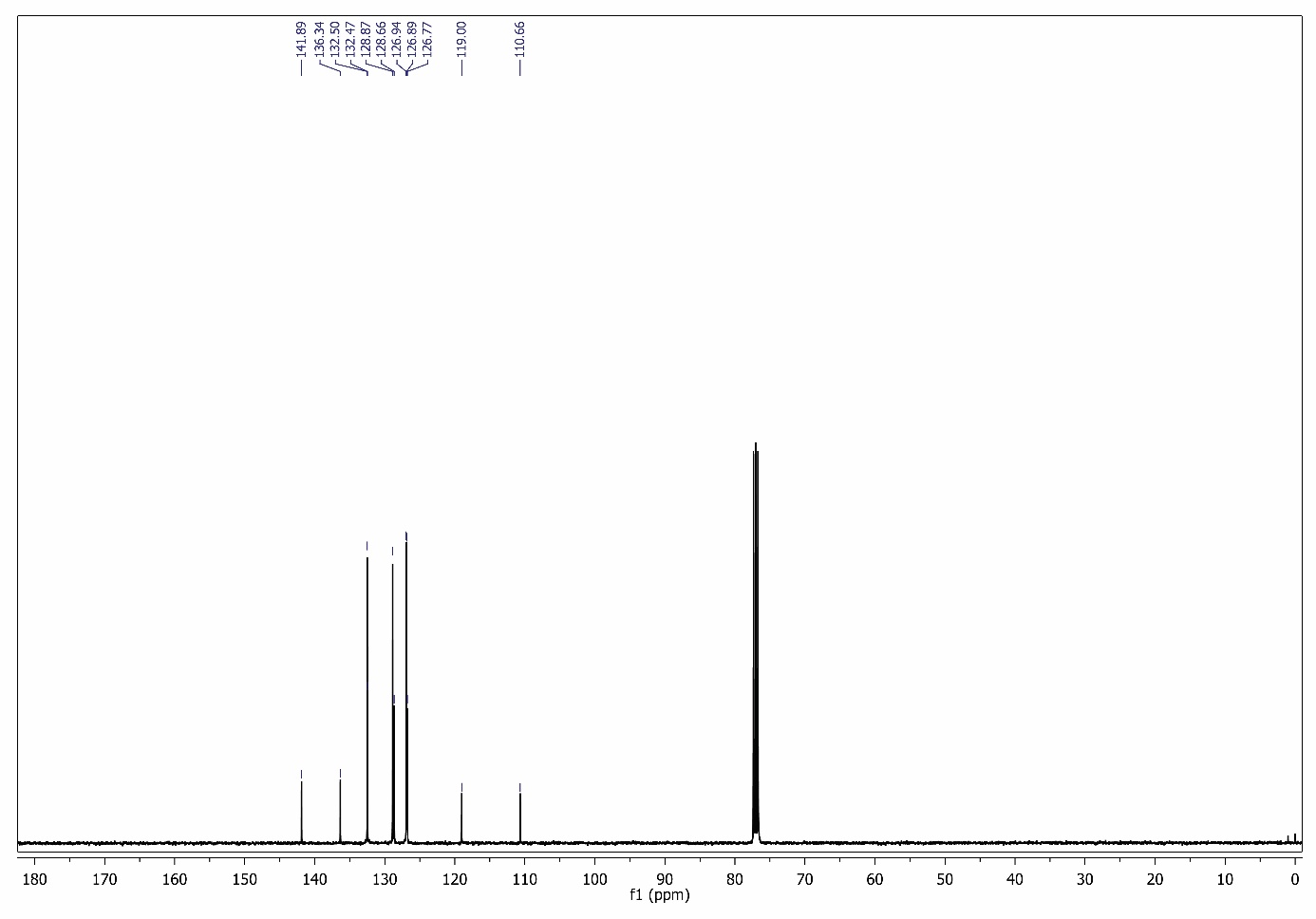


**Figure S16.** ^13^C NMR of (E)-4-styrylbenzonitrile.


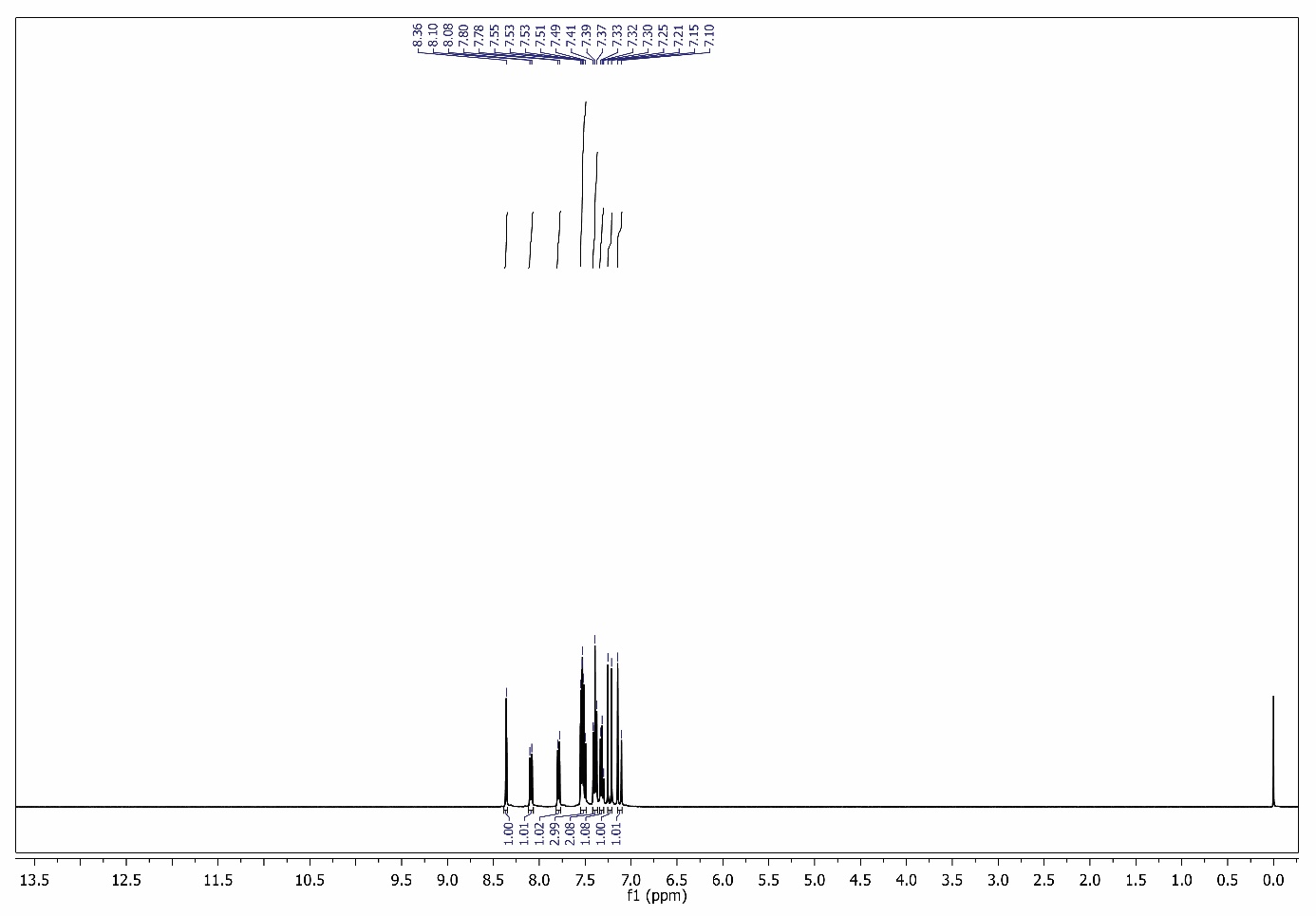


**Figure S17.** ^1^H NMR of (E)-1-nitro-3-styrylbenzene.


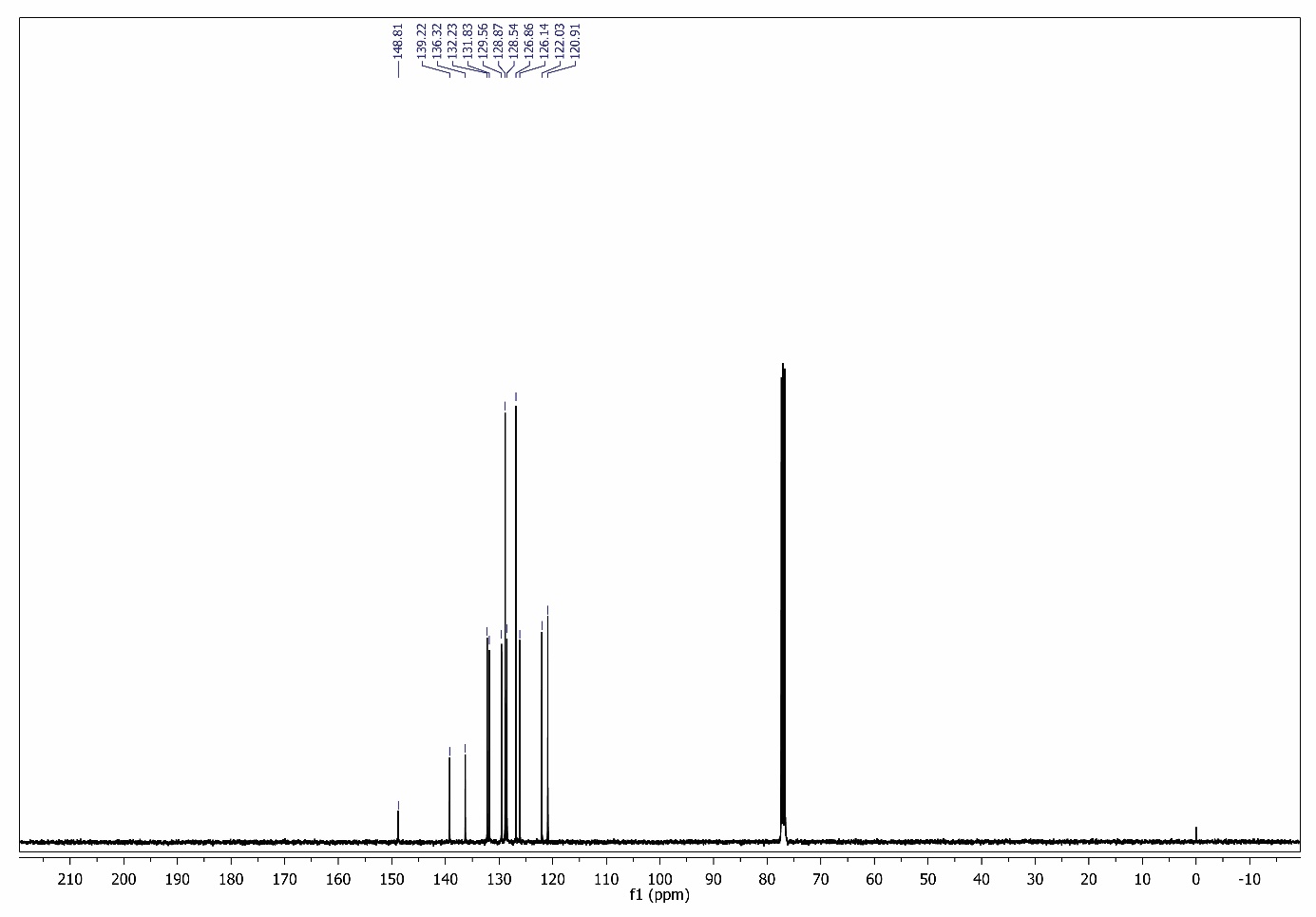


**Figure S18.** ^13^C NMR of (E)-1-nitro-3-styrylbenzene.

1. **E-mail address*: mahmoudnasr81@gmail.com (M. Nasrollahzadeh). [↑](#footnote-ref-1)
